# Net Benefit of Early Anticoagulation for Stroke With Atrial Fibrillation: Post Hoc Analysis of the ELAN Randomized Clinical Trial

**DOI:** 10.1001/jamanetworkopen.2024.56307

**Published:** 2025-01-28

**Authors:** Alexandros A. Polymeris, Mattia Branca, P. N. Sylaja, Else Charlotte Sandset, Diana Aguiar de Sousa, Götz Thomalla, Maurizio Paciaroni, Thomas Gattringer, Daniel Strbian, Sven Trelle, Patrik Michel, Krassen Nedeltchev, Leo H. Bonati, George Ntaios, Masatoshi Koga, Zuzana Gdovinova, Robin Lemmens, Natan M. Bornstein, Peter Kelly, Martina B. Goeldlin, Stefanie Abend, Magdy Selim, Mira Katan, Thomas Horvath, Jesse Dawson, Urs Fischer

**Affiliations:** 1 Department of Neurology and Stroke Center, University Hospital Basel and University of Basel, Switzerland; 2 Stroke Division, Department of Neurology, Beth Israel Deaconess Medical Center, Harvard Medical School, Boston, Massachusetts; 3 Department of Clinical Research, CTU Bern, University of Bern, Bern, Switzerland; 4 Sree Chitra Tirunal Institute for Medical Sciences and Technology, Thiruvananthapuram, India; 5 Department of Neurology, Oslo University Hospital, Norway; 6 The Norwegian Air Ambulance Foundation, Oslo, Norway; 7 Stroke Center, Lisbon Central University Hospital–ULS São José, Portugal; 8 Instituto de Medicina Molecular JLA, Faculdade de Medicina, Universidade de Lisboa, Portugal; 9 Department of Neurology, University Medical Center Hamburg–Eppendorf, Hamburg, Germany; 10 Internal, Vascular, and Emergency Medicine, Stroke Unit, Santa Maria della Misericordia Hospital, University of Perugia, Italy; 11 Clinical Neurology Unit, Department of Neuroscience and Rehabilitation, University of Ferrara, Ferrara, Italy; 12 Department of Neurology, Medical University of Graz, Graz, Austria; 13 Department of Neurology, Helsinki University Hospital and University of Helsinki, Helsinki, Finland; 14 Department of Neurology, University Hospital Lausanne, University of Lausanne, Switzerland; 15 Department of Neurology, Cantonal Hospital Aarau, Switzerland; 16 Research Department, Reha Rheinfelden, Rheinfelden, Switzerland; 17 Department of Internal Medicine, Faculty of Medicine, School of Health Sciences, University of Thessaly, Larissa, Greece; 18 Department of Cerebrovascular Medicine, National Cerebral and Cardiovascular Center, Osaka, Japan; 19 Department of Neurology, Faculty of Medicine, Pavol Jozef Safarik University and University Hospital Louis Pasteur, Košice, Slovakia; 20 Department of Neurosciences, Experimental Neurology, KU Leuven, Leuven, Belgium; 21 Department of Neurology, University Hospitals Leuven, Leuven, Belgium; 22 Department of Neurology, Shaare Zedek Medical Center, Jerusalem, Israel; 23 Department of Neurology, Mater Misericordiae University Hospital, Dublin, Ireland; 24 School of Medicine, University College Dublin, Dublin, Ireland; 25 Health Research Board Stroke Clinical Trials Network Ireland, Dublin, Ireland; 26 Department of Neurology, Inselspital, Bern University Hospital and University of Bern, Bern, Switzerland; 27 School of Cardiovascular and Metabolic Health, College of Medical, Veterinary & Life Sciences, Queen Elizabeth University Hospital, Glasgow, United Kingdom

## Abstract

**Importance:**

The net clinical effect of early vs later direct oral anticoagulant (DOAC) initiation after atrial fibrillation–associated ischemic stroke is unclear.

**Objective:**

To investigate whether early DOAC treatment is associated with a net clinical benefit (NCB).

**Design, Setting, and Participants:**

This was a post hoc analysis of the Early Versus Late Initiation of Direct Oral Anticoagulants in Post–Ischaemic Stroke Patients With Atrial Fibrillation (ELAN) open-label randomized clinical trial conducted across 103 sites in 15 countries in Europe, the Middle East, and Asia between November 6, 2017, and September 12, 2022, with a 90-day follow-up. Participants included patients with atrial fibrillation–associated acute ischemic stroke, excluding those with therapeutic anticoagulation at stroke onset or with severe hemorrhagic transformation of the ischemic infarct.

**Intervention:**

Early DOAC initiation (<48 hours after minor and moderate stroke, 6-7 days after major stroke) vs later initiation (3-4 days after minor stroke, 6-7 days after moderate stroke, and 12-14 days after major stroke).

**Main Outcomes and Measures:**

The main measure was the NCB of early treatment over later treatment, calculated by subtracting the weighted rate of excess bleeding events (major extracranial or intracranial hemorrhage) attributable to early treatment from the rate of excess ischemic events (recurrent stroke or systemic embolism) possibly prevented by early treatment within 30 days (main analysis) or 90 days (ancillary analysis). An established weighting scheme was used to account for the different clinical impact of bleeding relative to ischemic outcomes. Event rates were derived from adjusted logistic models. The analysis included all evaluable randomized ELAN participants.

**Results:**

Of the original 2013 ELAN participants, 1966 were eligible for analysis (977 [49.7%] assigned to early DOAC initiation, 989 [50.3%] assigned to later DOAC initiation; median [IQR] age 77 [70-84] years; 1075 [54.7%] male). The 30-day NCB of early treatment over later treatment ranged from 1.73 (95% CI, 0.06-3.40) to 1.72 (95% CI, −0.63 to 3.98) weighted events possibly prevented per 100 participants for intracranial hemorrhage weights 1.5 to 3.3. The 90-day NCB ranged from 2.16 (95% CI, 0.30-3.87) to 2.14 (95% CI, −0.26 to 4.41) weighted events per 100 participants.

**Conclusions and Relevance:**

This post hoc analysis of a randomized clinical trial estimated a sizeable NCB of early anticoagulation for patients after atrial fibrillation–associated ischemic stroke. Although estimates cannot exclude the possibility of no benefit or small net harm, the findings suggest that early treatment may be more favorable.

**Trial Registration:**

ClinicalTrials.gov Identifier: NCT03148457

## Key Points

**Question:**

Is early anticoagulation after atrial fibrillation–associated ischemic stroke associated with a net clinical benefit vs later treatment?

**Findings:**

This post hoc analysis of 1966 participants from the ELAN randomized trial balancing the benefit in reduction of ischemic outcomes (recurrent stroke or systemic embolism) against the risk of bleeding outcomes (major extracranial or intracranial hemorrhage) by weighting events for their different clinical importance estimated a net clinical benefit of approximately 2 weighted events per 100 persons possibly prevented with early treatment with direct oral anticoagulants; however, estimates cannot exclude the possibility of no benefit or small net harm.

**Meaning:**

Early anticoagulation may yield a sizeable net clinical benefit for patients after atrial fibrillation–associated acute ischemic stroke.

## Teaser

This post hoc analysis of a randomized clinical trial estimates the benefit in reduction of ischemic outcomes vs the risk of bleeding outcomes to assess whether early vs later initiation of direct oral anticoagulants is associated with a net clinical benefit for patients after atrial fibrillation–associated acute ischemic stroke.

## Introduction

After an acute ischemic stroke associated with atrial fibrillation, early direct oral anticoagulant (DOAC) initiation may reduce the risk of recurrence but expose patients to a higher risk of bleeding complications—particularly intracranial hemorrhage (ICH)—compared with delayed treatment initiation.^1^ Several observational studies and 3 recent randomized clinical trials investigated the safety and efficacy of early vs later DOAC initiation by comparing composite outcomes, which included recurrent ischemic stroke and bleeding events.^2,3,4,5^ The Timing of Oral Anticoagulant Therapy in Acute Ischemic Stroke With Atrial Fibrillation^4^ and the Optimal Timing of Anticoagulation After Acute Ischaemic Stroke^5^ randomized clinical trials demonstrated noninferiority of early treatment, but failed to show superiority. The Early Versus Late Initiation of Direct Oral Anticoagulants in Post–Ischaemic Stroke Patients With Atrial Fibrillation (ELAN) randomized clinical trial adopted no formal hypothesis testing and estimated the 30-day incidence of recurrent ischemic stroke, systemic embolism, major extracranial bleeding, ICH, or vascular death to be 1.2% lower (ranging from 2.8% lower to 0.5% higher) with early DOAC initiation.^3^ Taken together, in these trials, composite outcomes did not occur in excess and even seemed to be less frequent with early treatment.

However, merely adding up the number of ischemic and bleeding events may be less informative or even misleading, as their clinical significance is not equivalent.^6^ In fact, the disability and mortality of ICH tend to be greater compared with those of ischemic stroke,^7^ while the clinical impact of extracranial bleeding may be less severe. While the aforementioned studies^2,3,4,5^ identified no particular safety concerns, uncertainty remains about the net effect of the early vs later treatment approach.^8,9,10^ For a clinically meaningful assessment of the net effect of one approach over the other, not only the number of events but also their different clinical impact in terms of death and disability should be considered. In this post hoc analysis of the ELAN trial, we aimed to comprehensively investigate the net clinical benefit (NCB) of early vs later treatment as a single measure of the overall treatment effect, accounting for differences in the clinical importance between ischemic and bleeding events to better inform clinical practice.

## Methods

### Study Design and Participants

This is a post hoc analysis of the ELAN randomized clinical trial. The trial protocol, data collection methods, and main results have been published previously.^3,11^ In short, ELAN randomized participants with acute ischemic stroke and atrial fibrillation to early (<48 hours after minor and moderate stroke, 6-7 days after major stroke) vs later (3-4 days after minor stroke, 6-7 days after moderate stroke, and 12-14 days after major stroke) DOAC initiation in a 1:1 ratio across 103 stroke units and centers in 15 countries in Europe, the Middle East, and Asia between November 6, 2017, and September 12, 2022. Patients who received reperfusion therapies or antiplatelet treatment and patients with petechial hemorrhagic transformation of the infarcted brain tissue were eligible for participation, but patients with therapeutic anticoagulation at stroke onset or with more severe hemorrhagic transformation were excluded. Randomization was done with minimization for age (<70 or ≥70 years), infarct size (minor, moderate, or major), National Institutes of Health Stroke Scale (NIHSS) score at randomization (<10 or ≥10; scores range from 0-42, with higher scores indicating more severe stroke), and recruiting site. Minor infarcts were those of less than 1.5 cm, moderate were those greater than 1.5 cm in the anterior circulation but not involving the entire territory of the middle or anterior cerebral artery, and major were those involving the entire middle or anterior cerebral artery territory or those greater than 1.5 cm in the posterior circulation.^3,11,12^ Cardiovascular risk factors, comorbidities, and details on DOAC treatment were collected at baseline, and participants were followed up at 30 and 90 days after randomization for the outcomes outlined below. These outcomes were defined as in the main trial and centrally adjudicated in a blinded manner.^3,11^ All study data were gathered by local investigators and collected in a web-based database hosted by the Clinical Trial Unit, University of Bern, Bern, Switzerland. The statistical analysis plan for this post hoc analysis is provided in Supplement 1. The ELAN protocol was approved by all responsible ethics committees and, if applicable, by the regulatory authorities in the countries in which the trial was conducted. The participant, next of kin or another legal representative, or an independent physician provided written informed consent before enrollment, according to country-specific requirements. ELAN was conducted in accordance with the Good Clinical Practice guidelines of the International Council for Harmonisation E6 requirements and the Declaration of Helsinki.^13^ Herein, we included all evaluable randomized ELAN participants as in the main trial analysis.^3^ This study followed the Consolidated Standards of Reporting Trials (CONSORT) reporting guideline.

### Outcomes

For the main NCB analysis of this study, we considered the following outcomes at 30 days, in keeping with the main trial analysis^3,11^: (1) recurrent ischemic stroke (defined as evidence of acute cerebral infarction with neuroimaging or as a clinical diagnosis with symptoms lasting longer than 24 hours and exclusion of other causes through neuroimaging), (2) systemic embolism (SE; defined as clinical or radiologic evidence of abrupt arterial occlusion of an extremity or organ other than the brain in the absence of another likely mechanism), (3) major extracranial bleeding (defined as fatal, life-threatening, or clinically overt hemorrhage associated with a hemoglobin level decrease of ≥2 g/dL [to convert to g/L, multiply by 10.0] over a 24-hour period, transfusion of ≥2 units of packed red cells, or occurring in a critical body part), and (4) symptomatic ICH (defined as subdural, epidural, subarachnoid, or intracerebral hemorrhage leading to clinical symptoms, hospitalization, or death). In ancillary analyses we also considered these outcomes at 90 days and the additional outcome of nonmajor bleeding (ie, not satisfying the aforementioned criteria for major bleeding).

### Statistical Analysis

We categorized participants according to allocation into an early anticoagulation or later anticoagulation group according to a modified intention-to-treat strategy, as in the main trial.^3^ We present baseline variables in both groups using descriptive statistics, that is, frequencies and percentages for categorical data, and the median and IQR for continuous data, and report the rate of missing values. Statistical analyses were performed using R, version 4.3.1 (R Project for Statistical Computing).

In the main analysis, we examined the NCB of early vs later DOAC initiation at 30 days, adopting established methods as in prior research,^6,14,15,16,17^ with weighting of the type of events for their impact on death and disability relative to recurrent ischemic stroke. For this, we calculated the NCB by subtracting the rate of excess bleeding events attributable to early treatment from the rate of excess ischemic events possibly prevented by early treatment, according to the following formula:

*NCB* = (*RI_L_* − *RI_E_*) + [SE_w_ × (*RSE_L_* − *RSE_E_*)] − [ICH_w_ × (*RICH_E_* − *RICH_L_*)] − [MB_w_ × (*RMB_E_* − *RMB_L_*)],

where *RI_L_* is the rate of recurrent ischemic stroke for late DOAC; *RI_E_*, the rate of recurrent ischemic stroke for early DOAC; *RSE_L_*, the rate of SE for late DOAC; *RSE_E_*, the rate of SE for early DOAC; *RICH_E_*, the rate of ICH for early DOAC; *RICH_L_*, the rate of ICH for late DOAC; *RMB_E_*, the rate of major extracranial bleeding for early DOAC; and *RMB_L_*, the rate of major extracranial bleeding for late DOAC. Weight values are derived from previous empirical research,^6,14,15,16^ with ischemic stroke being assigned a weight of 1.0 (reference event), SE a weight of 0.9 (SE_w_), and major extracranial bleeding a weight of 0.7 (MB_w_), while the weight for ICH (ICH_w_) varies from 1.5 to 3.3. We performed the NCB analysis across the entire range of ICH weights, as in prior research.^17,18^ Deviating from our original analysis plan, which combined recurrent ischemic stroke and systemic embolism into a single reference ischemic outcome, we used the method of Eikelboom et al^15^ to distinguish between the 2 ischemic outcomes, as the most appropriate approach for our data.

We extracted the outcome event rates from a Firth logistic regression model for each outcome, as in the primary analysis of the main trial. We adjusted the models for the minimization factors age, NIHSS score, and infarct size, all as categorical variables, as described above, in keeping with the main trial analysis. Event rates indicate the adjusted proportion of participants that experienced an outcome at least once. In this analysis, we considered all participants with evaluable follow-up data except for those with death (from any cause) as first outcome, who were excluded from the logistic models. Participants who withdrew consent or were unavailable for follow-up were included if they experienced evaluable events; otherwise, they were excluded from the analysis.

We report the NCB in weighted events per 100 participants along with 95% CIs, calculated based on 1000 bootstrap replications. Positive NCB values represent favor for early DOAC initiation over later DOAC initiation, while negative values indicate net harm associated with early treatment. We also provided estimates for the number needed to treat (NNT) early as opposed to later to prevent 1 weighted event (calculated as NNT = 1 / NCB, as in prior research^19^).

We additionally performed the following ancillary analyses:

1. Calculation of the NCB of early vs later DOAC initiation at 90 days, using the same method as in the main analysis but considering all outcomes within 90 days instead of 30 days.
2. Calculation of the NCB of early vs later DOAC initiation at 30 days according to infarct size, that is, separately in the subgroups with minor, moderate, or major stroke. Here, we adjusted the logistic models out of which the event rates were derived only for age and NIHSS score.
3. Calculation of the NCB of early vs later DOAC initiation at 30 days including nonmajor bleeding (non-MB) events using the same method as in the main analysis and according to the following formula, where the non-MB weight varies from 0.1 to 0.5, as in previous research^18^:
  *NCB* = (*RI_L_* − *RI_E_*) + [SE_w_ × (*RSE_L_* − *RSE_E_*)] − [ICH_w_ × (*RICH_E_* − *RICH_L_*)] − [MB_w_ × (*RMB_E_* − *RMB_L_*)]
  − [non-MB_w_ × (*Rnon-MB_E_* − *Rnon-MB_L_*)].

## Results

### Main NCB Analysis at 30 Days

**Figure 1.**
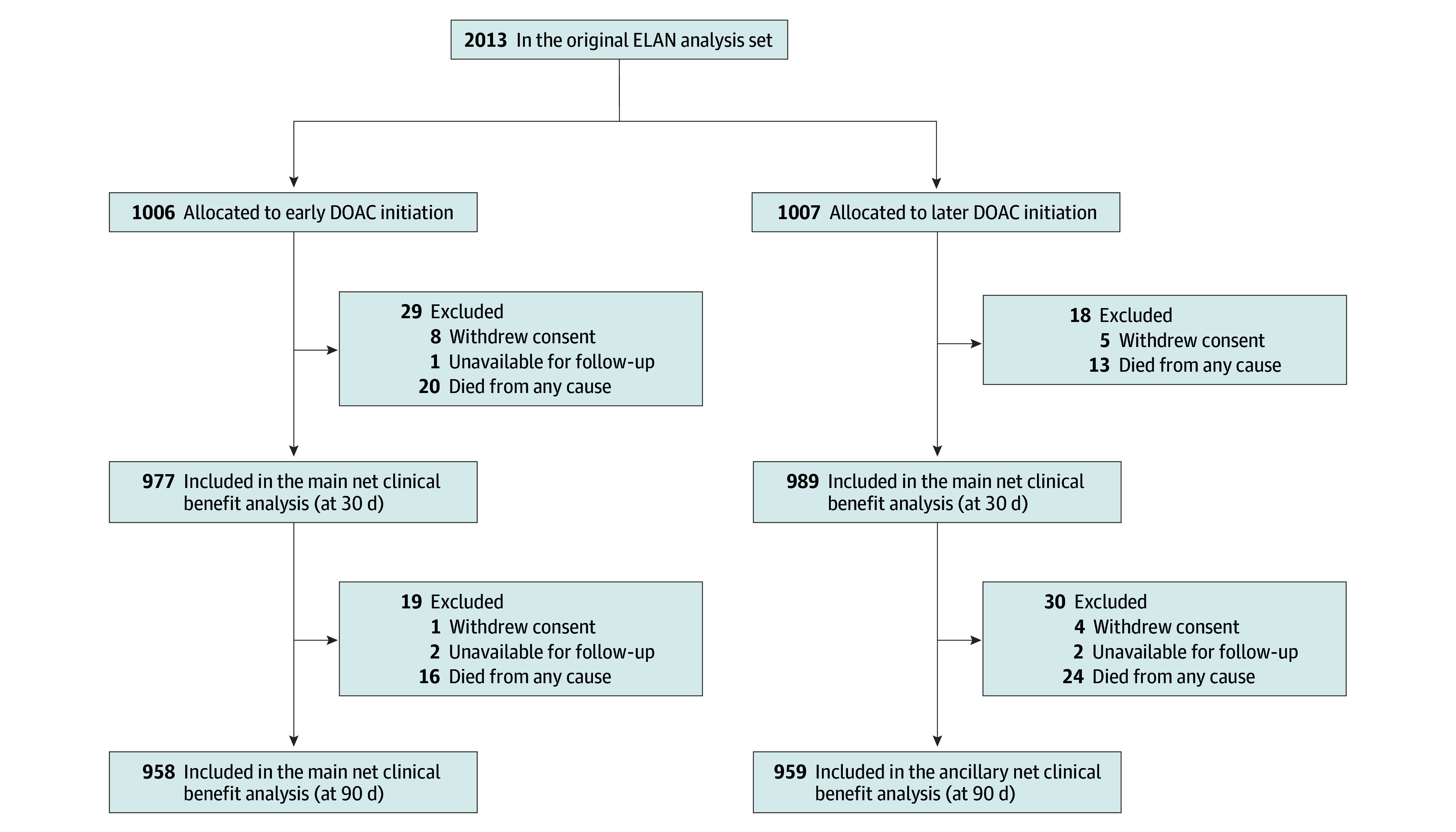
Study Flowchart DOAC indicates direct oral anticoagulant; ELAN, the Early Versus Late Initiation of Direct Oral Anticoagulants in Post–Ischaemic Stroke Patients With Atrial Fibrillation randomized clinical trial.

Out of the full ELAN dataset of 2013 participants,^3^ 1966 (median [IQR] age 77 [70-84] years; 891 [45.3%] female and 1075 [54.7%] male) were included in the main 30-day NCB analysis, after exclusion of 13 participants who withdrew consent, 1 participant who was unavailable for follow-up, and 33 participants with death of any cause as the first event. The study flowchart is shown in Figure 1. As in the main trial, baseline data were well balanced between the early and later treatment groups in the main NCB analysis, and the variables adjusted for in the models were complete (Table 1).

**Table 1.** Baseline Characteristics of Participants Included in the Main NCB Analysis at 30 Days

Characteristic	No./total No. (%)	
	Participants with early DOAC initiation (n = 977)	Participants with later DOAC initiation (n = 989)
Age, median (IQR), y	77 (70-84)	78 (70-84)
Sex, No. (%)		
Female	446 (45.6)	445 (45.0)
Male	531 (54.4)	544 (55.0)
Stroke severity according to infarct size, No. (%)^a^		
Minor	372 (38.1)	368 (37.2)
Moderate	389 (39.8)	393 (39.7)
Major	216 (22.1)	228 (23.1)
NIHSS score, median (IQR)^b^	3 (1-6)	3 (1-6)
CHA_2_DS_2_-VASc score, median (IQR)^c^	5 (4-6)	5 (4-6)
History of		
Ischemic stroke or TIA	155/967 (16.0)	172/984 (17.5)
Systemic embolism	18/967 (1.9)	29/982 (3.0)
Hypertension	671/967 (69.4)	660/981 (67.3)
Myocardial infarction	78/967 (8.1)	84/979 (8.6)
Heart failure	62/911 (6.8)	59/923 (6.4)
Peripheral artery disease	33/944 (3.5)	47/957 (4.9)
Diabetes	179/967 (18.5)	156/984 (15.9)
Dyslipidemia	427/947 (45.1)	418/963 (43.4)
Current or past smoking	246/925 (26.6)	240/930 (25.8)
Creatinine clearance, median (IQR), mL/min/1.73 m^2^	71 (60-86)	69 (57-86)
mRS score before stroke ≤2^d^	867/976 (88.8)	884/988 (89.5)
Acute reperfusion therapy		
Intravenous thrombolysis	204/957 (21.3)	230/969 (23.7)
Thrombectomy	383/957 (40.0)	374/969 (38.6)
DOAC type		
Once daily	194/971 (20.0)	203/977 (20.8)
Twice daily	777/971 (80.0)	774/977 (79.2)
DOAC dose according to SPCs		
Full dose	795/974 (81.6)	797/983 (81.1)
Reduced dose	179/974 (18.4)	186/983 (18.9)

Abbreviations: CHA_2_DS_2_-VASc, congestive heart failure, hypertension, age 75 years or older, diabetes, stroke or TIA, vascular disease, age 65 to 74 years, and female sex; DOAC, direct oral anticoagulant; mRS, modified Rankin Scale; NCB, net clinical benefit; NIHSS, National Institutes of Health Stroke Scale score at randomization; SPCs, summary of product characteristics; TIA, transient ischemic attack.

SI conversion factor: To convert creatinine clearance to mL/s/m^2^, multiply by 0.0167.

^a^ Minor infarcts were <1.5 cm; moderate were >1.5 cm in the anterior circulation but not involving the entire territory of the middle or anterior cerebral artery; and major involved the entire middle or anterior cerebral artery territory or were >1.5 cm in the posterior circulation.

^b^ NIHSS scores range from 0-42, with higher scores indicating more severe stroke.

^c^ CHA_2_DS_2_-VASc scores range from 0 to 9, with higher values indicating higher stroke risk.

^d^ Modified Rankin Scale scores range from 0 (no symptoms) to 6 (death).

**Figure 2.**
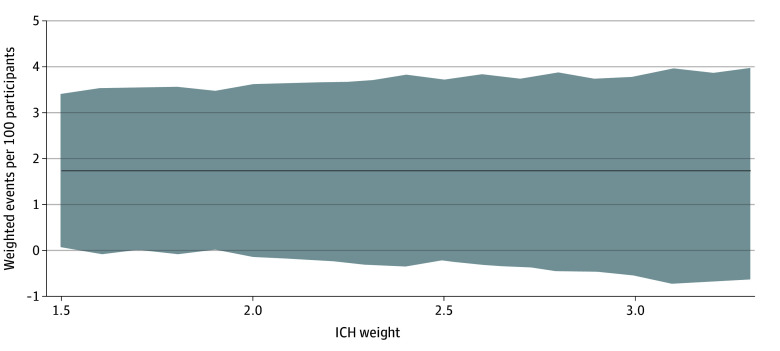
Net Clinical Benefit of Early Direct Oral Anticoagulant (DOAC) Initiation Over Later DOAC Initiation at 30 Days Net clinical benefit is expressed in weighted events possibly prevented per 100 participants. The solid line represents the net clinical benefit estimate across the entire range of intracranial hemorrhage (ICH) weights; and the gray shaded area, 95% Cl.

Among 1966 participants, we observed 39 recurrent ischemic strokes, 13 SE events, 8 major extracranial hemorrhages, and 4 symptomatic ICH events at 30 days. No participant had multiple occurrences of any given outcome. All 30-day events according to treatment group are shown in Table 2. The point estimates for the NCB of early DOAC initiation over later DOAC initiation at 30 days were consistently positive (ie, in favor of early treatment) across the entire range of ICH weights used (1.73 [95% CI, 0.06-3.40] to 1.72 [95% CI, −0.63 to 3.98] weighted events per 100 participants possibly prevented with early DOAC initiation for ICH weights 1.5 to 3.3), with wide CIs, the lower limit of which was close to the null at all weights, mostly crossing it (Figure 2). The corresponding NNT was 58. The detailed results of the main NCB analysis are presented in eTable 1 in Supplement 2.

**Table 2.** Thirty-Day Outcomes According to Treatment Allocation

Outcome	Participants, No. (%)		
	Total (N = 1966)	Early DOAC initiation (n = 977)	Later DOAC initiation (n = 989)
Recurrent ischemic stroke	39 (2.0)	14 (1.4)	25 (2.5)
Systemic embolism	13 (0.7)	4 (0.4)	9 (0.9)
Major extracranial bleeding	8 (0.4)	3 (0.3)	5 (0.5)
Symptomatic intracranial hemorrhage	4 (0.2)	2 (0.2)	2 (0.2)
Nonmajor bleeding	56 (2.8)	29 (3.0)	27 (2.7)

Abbreviation: DOAC, direct oral anticoagulant.

### Ancillary NCB Analyses

#### NCB At 90 Days

**Figure 3.**
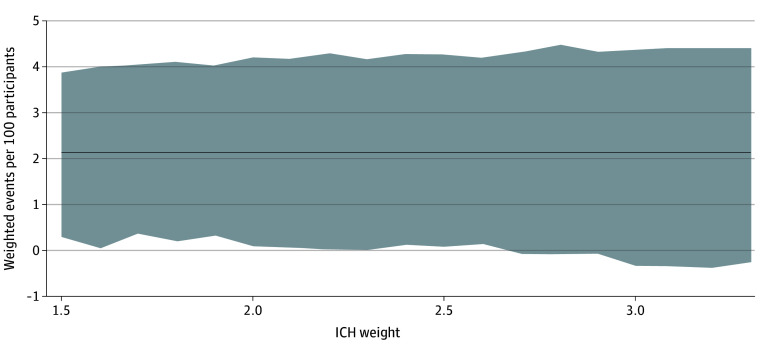
Net Clinical Benefit of Early Direct Oral Anticoagulant (DOAC) Initiation Over Later DOAC Initiation at 90 Days Net clinical benefit is expressed in weighted events possibly prevented per 100 participants. The solid line represents the net clinical benefit estimate across the entire range of intracranial hemorrhage (ICH) weights; and the gray shaded area, 95% CIs.

A total of 1917 participants were included in the ancillary 90-day NCB analysis (Figure 1). As in the main analysis, their baseline characteristics were balanced between the groups (eTable 2 in Supplement 2). At 90 days, we recorded 48 recurrent ischemic strokes, 14 SE events, 11 major extracranial hemorrhages and 4 symptomatic ICH events (eTable 3A in Supplement 2). No participant had multiple occurrences of any given outcome. Consistent with the main analysis, the point estimates for the NCB of early DOAC initiation over later DOAC initiation at 90 days were positive regardless of the ICH weight used (2.16 [95% CI, 0.30-3.87] to 2.14 [95% CI, −0.26 to 4.41] weighted events per 100 participants possibly prevented with early DOAC initiation for ICH weights 1.5 to 3.3), with 95% CIs above the null for ICH weights 2.6 or lower but crossing it at higher ICH weights (Figure 3; eTable 4 in Supplement 2). The corresponding NNT was 47.

#### NCB at 30 Days According to Infarct Size

eTable 3B in Supplement 2 gives all 30-day events in subgroups according to treatment allocation and infarct size. Repeating the main 30-day NCB analysis separately in subgroups according to infarct size showed no evidence of a nonneutral net effect of early DOAC initiation over later DOAC initiation for participants with minor or moderate stroke but a strong signal for a positive NCB in participants with major stroke (eFigure 1, eTable 5 in Supplement 2).

#### NCB at 30 Days Including Nonmajor Bleeding Events

Among the 1966 participants included in the main analysis, we observed a total of 56 nonmajor bleeding events within 30 days (Table 2). Repeating the main NCB analysis after including nonmajor bleeding yielded consistent results with positive NCB estimates regardless of the weights used for ICH and nonmajor bleeding, with wide 95% CIs crossing the null line (eFigure 2, eTable 6 in Supplement 2).

## Discussion

This post hoc analysis of the ELAN randomized clinical trial investigated the weighted NCB of early DOAC initiation over later DOAC initiation after acute ischemic stroke in people with atrial fibrillation. Using a neuroimaging-based approach to guide treatment timing and established methodology to calculate NCB, we estimated that early treatment initiation possibly prevented about 1.7 weighted events per 100 persons within 30 days, a sizable net benefit with an NNT of 58. However, considering the width of the 95% CIs for the estimates, this finding is statistically compatible with the possibility of a neutral net effect or small net harm. When considering events within 90 days, early treatment was estimated to confer an even larger net benefit by preventing approximately 2.1 weighted events (NNT of 47) but with 95% CIs not completely excluding the possibility of no benefit or a very small net harm. These findings suggest that early DOAC initiation may be more favorable than later treatment.

Our findings are in line with the main analysis of ELAN^3^ as well as with other randomized and observational data showing that composite outcomes adding up the number of ischemic and hemorrhagic events do not occur in excess but may even be less frequent with early treatment, without particular safety concerns.^2,4,5,20^ Expanding on these results, the present study examined the NCB as a single measure of the global treatment effect that balances the benefit in ischemic stroke reduction against the weighted risk of bleeding complications, and provides new evidence in favor of early treatment. Weighting is important because not all types of ischemic and hemorrhagic events are equally disabling or deadly but rather differ in their clinical significance. This is reflected in the NCB weights we used, which account for the different impact of the harmful events that early treatment may prevent (such as ischemic stroke or SE) and those it may cause (such as ICH or other bleeding). Reassuringly, our results were consistent across a broad spectrum of weights used for bleeding events and regardless of whether nonmajor bleeding was considered in the NCB calculations.

Of note, our subgroup analysis according to infarct size indicated that early treatment may exert most benefit in participants with major stroke. This finding is consistent with a previous subgroup analysis of ELAN^12^ but should be interpreted with caution considering the generally low number of outcome events in the subgroups—particularly the scarce ICH events, which were the most heavily weighted ones and may drive these subgroup NCB results.

### Strengths and Limitations

Our study has several strengths. First, we investigated the question of early treatment vs late treatment by applying established NCB methodology to reanalyze high-quality randomized data from ELAN, one of the largest trials investigating this issue. Our approach may enhance the interpretability of ELAN, which was designed to provide treatment effect estimates without hypothesis testing. Second, we used several sensitivity analyses in our NCB calculations, which all resulted in consistent findings.

We acknowledge the following limitations. First, this analysis, although done according to a predefined statistical analysis plan, was not specified in the original ELAN protocol but was developed post hoc. Second, the generally low numbers of events resulted in imprecise NCB estimates, as reflected in their wide 95% CIs. Because of this, the possibility of a neutral net effect or small net harm cannot be ruled out. Third, although the weighting schemes used in our NCB calculations were derived from large, high-quality datasets and successfully applied in previous research,^6,14,15,16^ they may not reflect the contemporary relative clinical importance of the investigated outcomes, as recent advances in stroke treatment may have modified this. Similarly, our approach did not differentiate between milder and more severe strokes, which are known to carry different prognoses.^21^ Ideally, future research should establish new and more nuanced weights reflecting modern stroke outcomes. Fourth, by definition, the NCB calculations did not include death when this was the first outcome to occur. NCB analyses may therefore be of limited usefulness when death as first event without other preceding outcome events is much more common with one of the compared treatment approaches than the other. Importantly, this was not the case in ELAN (Figure 1), but the small number of events for some of the outcomes disallowed the use of competing risk survival analysis, which was originally specified in our predefined analysis plan as an alternative method to obtain event rates for an ancillary NCB analysis.

## Conclusions

In this post hoc analysis of a randomized clinical trial, we estimated that early DOAC initiation yielded a sizeable NCB by preventing approximately 2 weighted events per 100 persons after atrial fibrillation–associated ischemic stroke. Although estimates could not exclude the possibility of no benefit or small net harm, our findings lend further support to the early treatment approach.

## Notes

Supplement 1.
Statistical Analysis Plan

Supplement 2. **eTable 1.** Net clinical benefit of early over later DOAC initiation at 30 days **eTable 2.** Baseline characteristics of participants included in the ancillary NCB analysis at 90 days **eTable 3.** 90-Day outcomes according to treatment allocation and 30-day outcomes according to treatment allocation and infarct size **eTable 4.** Net clinical benefit of early over later DOAC initiation at 90 days **eTable 5.** Net clinical benefit of early over later DOAC initiation at 30 days in subgroups according to infarct size **eTable 6.** Net clinical benefit of early over later DOAC initiation at 30 days including non-major bleeding with a weight of 0.1 and 0.5 **eFigure 1.** Net clinical benefit of early over later DOAC initiation at 30 days in weighted events possibly prevented per 100 participants (solid line) with 95% confidence intervals (grey shaded area) in subgroups according to infarct size **eFigure 2.** Net clinical benefit of early over later DOAC initiation at 30 days in weighted events possibly prevented per 100 participants (solid line) with 95% confidence intervals (grey shaded area), including non-major bleeding events with a weight of 0.1 and 0.5

Supplement 3. **Nonauthor Collaborators.** The ELAN Investigators

Supplement 4.
Data Sharing Statement
