# Supplementary material for: Net Benefit of Early Anticoagulation for Stroke With Atrial Fibrillation: Post Hoc Analysis of the ELAN Randomized Clinical Trial
Source: JAMA Netw Open. 2025 Jan 28;8(1):e2456307. doi: 10.1001/jamanetworkopen.2024.56307 (PMC11775740; doi:10.1001/jamanetworkopen.2024.56307)
Supplement: Supplement 1. — Statistical Analysis Plan [file jamanetwopen-e2456307-s001.pdf]

May 3, 2024

Early versus Late initiation of direct oral Anticoagulants in post-ischemic stroke patients with atrial fibrillatioN (ELAN): A Randomized Controlled Trial

Subanalysis Proposal and Analysis Plan:

Net clinical benefit of early versus late DOAC initiation in patients with ischemic stroke associated with atrial fibrillation – an analysis from the ELAN trial

1. Inclusion / Exclusion Criteria:

This analysis will include all participants from the main ELAN paper (i.e., modified intention-to-treat population; 2013 ELAN participants).[1]

2. Background, aims and hypothesis

Background and aims:

After an acute ischemic stroke associated with atrial fibrillation (AF), early initiation of direct oral anticoagulants (DOAC) may reduce the risk of recurrence but expose patients to a higher risk of bleeding complications compared to delayed treatment initiation. Recent randomized trials including ELAN investigated the safety and efficacy of early versus late DOAC treatment by comparing combined outcomes, which included recurrent ischemic stroke and bleeding events.[1, 2] Although combined outcomes were less frequent with early treatment, merely adding up the number or ischemic and bleeding events may be misleading,[1, 2] as their clinical significance is not equivalent.[3] In the absence of a nuanced risk-benefit assessment, uncertainty still remains about whether early treatment should be favored in clinical practice.

For a clinically meaningful assessment of the net clinical benefit (NCB) of the early over the late treatment approach, not only the number of events, but also their impact on death and disability should be considered. Here, we aim to comprehensively evaluate the NCB of early over late DOAC initiation in the ELAN trial, accounting for differences in the clinical importance between ischemic and bleeding events, in order to better inform clinical practice.

Hypothesis:

We hypothesize that the net clinical effect of early over late DOAC initiation is beneficial.

3. Endpoints

To calculate the NCB at 30 days (primary analysis) we will consider the following outcome events within 30d, defined as in the main analysis of the ELAN trial: (1) recurrent ischemic stroke, (2) systemic embolism, (3) major extracranial bleeding, (4) symptomatic intracranial hemorrhage.

Ancillary analyses will include the calculation of NCB at 90 days, for which the same outcomes as above (but within 90d) will be used, and the addition of nonmajor bleeding (defined as in the main trial) into the NCB calculation.

4. Analysis

Primary analysis: NCB within 30 days

We will calculate the net clinical benefit (NCB) of early over late DOAC initiation adopting established methodology as in prior research,[4, 5] with weighting of the type of events for their impact on death and disability relative to recurrent ischaemic stroke or systemic embolism. For this, we will calculate the NCB by subtracting the weighted rate of excess bleeding events attributable to early treatment from the rate of excess ischemic events prevented by early treatment, according to the following formula:

$$NCB = (R_{ischemic\ [lateDOAC]} - R_{ischemic\ [early\ DOAC]}) - weight_{ICH} * (R_{ICH\ [earlyDOAC]} - R_{ICH\ [lateDOAC]}) - weight_{major\ bleed} * (R_{major\ bleed\ [earlyDOAC]} - R_{major\ bleed\ [lateDOAC]})$$

where $R_{ischaemic}$ is the rate of recurrent ischaemic stroke or systemic embolism, R_{ICH} the rate of ICH, and $R_{major\ bleed}$ the rate of major extracranial bleeding. Weight values are derived from the literature,[3, 5, 6] with major extracranial bleeding being assigned a weight of 0.7 ($weight_{major\ bleed}$), while the weight for ICH varies from 1.5 to 3.1 ($weight_{ICH}$). We will perform the NCB analysis across the entire range of ICH weights, as in prior research.[4, 7]

The outcome event rates will be extracted from a Firth's logistic regression model, as in the main ELAN paper. The model will be adjusted for the stratification factors age (dichotomized to <70 years or ≥70 years), NIHSS (dichotomized to <10 or ≥10, and infarct size (minor, moderate, or major), in keeping with the main ELAN paper. Participants with death (of any cause, vascular or nonvascular) as first outcome will be excluded from the logistic model.

We will report the NCB in events per 100 person-months along with 95% CI, calculated based on 1,000 bootstrap replications.

Ancillary analyses:

A. NCB within 90 days

Analyze as in the primary analysis, but consider all outcomes within 90 days.

B. NCB (within 30 days) in the subgroups with minor – moderate – major stroke

Run the NCB analysis as in the primary analysis separately in the 3 subgroups according to infarct size. In this sensitivity analysis, the logistic models out of which the event rates will be extracted will be adjusted only for age and NIHSS.

C. NCB (within 30 days) including nonmajor bleeding

Analyze the NCB as in the primary analysis but also include a term for nonmajor bleeding (according to the formula below).

$$NCB = (R_{ischemic\ [lateDOAC]} - R_{ischemic\ [early\ DOAC]}) - weight_{ICH} * (R_{ICH\ [earlyDOAC]} - R_{ICH\ [lateDOAC]}) - weight_{major\ bleed} * (R_{major\ bleed\ [earlyDOAC]} - R_{major\ bleed\ [lateDOAC]}) - weight_{nonmajor\ bleed} * (R_{nonmajor\ bleed\ [earlyDOAC]} - R_{nonmajor\ bleed\ [lateDOAC]})$$

The value of the $weight_{nonmajor\ bleed}$ may vary between 0.1 to 0.5, as in previous research.[7]

D. NCB (within 30 days) using survival analysis to model the event rates

Analyze as in the primary analysis, but use survival analysis to calculate event rates. Here, the event rates indicate the rate of events / number of person-months observed and will be extracted from cause-specific cox proportional hazards regression models, in keeping with the main ELAN paper. The models will be adjusted for the stratification factors age (dichotomized to <70 years or ≥70 years), NIHSS (dichotomized to <10 or ≥10, infarct size (minor, moderate, or major), as in the main ELAN paper. Contrary to main analysis which will use logistic models to calculate the event rates, participants with death (of any cause) as first outcome will be included in this sensitivity analysis, as their observation time (up to the point of death, at which they will censored) will contribute to the total observed at-risk time.

References

1. Fischer U, Koga M, Strbian D, Branca M, Abend S, Trelle S, et al. Early versus later anticoagulation for stroke with atrial fibrillation. *N Engl J Med*. 2023
2. Oldgren J, Åsberg S, Hijazi Z, Wester P, Bertilsson M, Norrving B. Early versus delayed non-vitamin k antagonist oral anticoagulant therapy after acute ischemic stroke in atrial fibrillation (timing): A registry-based randomized controlled noninferiority study. *Circulation*. 2022;146:1056-1066
3. Connolly SJ, Eikelboom JW, Ng J, Hirsh J, Yusuf S, Pogue J, et al. Net clinical benefit of adding clopidogrel to aspirin therapy in patients with atrial fibrillation for whom vitamin k antagonists are unsuitable. *Ann Intern Med*. 2011;155:579-586
4. Polymeris AA, Macha K, Paciaroni M, Wilson D, Koga M, Cappellari M, et al. Oral anticoagulants in the oldest old with recent stroke and atrial fibrillation. *Ann Neurol*. 2022;91:78-88
5. Lip GY, Skjoth F, Nielsen PB, Larsen TB. Non-valvular atrial fibrillation patients with none or one additional risk factor of the cha2ds2-vasc score. A comprehensive net clinical benefit analysis for warfarin, aspirin, or no therapy. *Thromb Haemost*. 2015;114:826-834
6. Singer DE, Chang Y, Fang MC, Borowsky LH, Pomernacki NK, Udaltsova N, et al. The net clinical benefit of warfarin anticoagulation in atrial fibrillation. *Ann Intern Med*. 2009;151:297-305
7. Pan Y, Elm JJ, Li H, Easton JD, Wang Y, Farrant M, et al. Outcomes associated with clopidogrel-aspirin use in minor stroke or transient ischemic attack: A pooled analysis of clopidogrel in high-risk patients with acute non-disabling cerebrovascular events (chance) and platelet-oriented inhibition in new tia and minor ischemic stroke (point) trials. *JAMA Neurol*. 2019;76:1466-1473
