# Supplementary material for: Net Benefit of Early Anticoagulation for Stroke With Atrial Fibrillation: Post Hoc Analysis of the ELAN Randomized Clinical Trial
Source: JAMA Netw Open. 2025 Jan 28;8(1):e2456307. doi: 10.1001/jamanetworkopen.2024.56307 (PMC11775740; doi:10.1001/jamanetworkopen.2024.56307)
Supplement: Supplement 2. — eTable 1. Net clinical benefit of early over later DOAC initiation at 30 days eTable 2. Baseline characteristics of participants included in the ancillary NCB analysis at 90 days eTable 3. 90-Day outcomes according to treatment allocation and 30-day outcomes according to treatment allocation and infarct size eTable 4. Net clinical benefit of early over later DOAC initiation at 90 days eTable 5. Net clinical benefit of early over later DOAC initiation at 30 days in subgroups according to infarct size eTable 6. Net clinical benefit of early over later DOAC initiation at 30 days including non-major bleeding with a weight of 0.1 and 0.5 eFigure 1. Net clinical benefit of early over later DOAC initiation at 30 days in weighted events possibly prevented per 100 participants (solid line) with 95% confidence intervals (grey shaded area) in subgroups according to infarct size eFigure 2. Net clinical benefit of early over later DOAC initiation at 30 days in weighted events possibly prevented per 100 participants (solid line) with 95% confidence intervals (grey shaded area), including non-major bleeding events with a weight of 0.1 and 0.5 [file jamanetwopen-e2456307-s002.pdf]

Supplementary Online Content

Polymeris AA, Branca M, Sylaja PN, et al. Net benefit of early anticoagulation for stroke with atrial fibrillation: post hoc analysis of the ELAN randomized clinical trial. *JAMA Netw Open*. 2024;8(1):e2456307. doi:10.1001/jamanetworkopen.2024.56307

eTable 1. Net clinical benefit of early over later DOAC initiation at 30 days

eTable 2. Baseline characteristics of participants included in the ancillary NCB analysis at 90 days

eTable 3. 90-Day outcomes according to treatment allocation and 30-day outcomes according to treatment allocation and infarct size

eTable 4. Net clinical benefit of early over later DOAC initiation at 90 days

eTable 5. Net clinical benefit of early over later DOAC initiation at 30 days in subgroups according to infarct size

eTable 6. Net clinical benefit of early over later DOAC initiation at 30 days including non-major bleeding with a weight of 0.1 and 0.5

eFigure 1. Net clinical benefit of early over later DOAC initiation at 30 days in weighted events possibly prevented per 100 participants (solid line) with 95% confidence intervals (grey shaded area) in subgroups according to infarct size

eFigure 2. Net clinical benefit of early over later DOAC initiation at 30 days in weighted events possibly prevented per 100 participants (solid line) with 95% confidence intervals (grey shaded area), including non-major bleeding events with a weight of 0.1 and 0.5

This supplementary material has been provided by the authors to give readers additional information about their work.

© 2025 Polymeris AA et al. *JAMA Network Open*.

eTable 1. Net clinical benefit of early over later DOAC initiation at 30 days

ICH weight	weighted rate of events* in participants with early DOAC initiation (95% CI)	weighted rate of events* in participants with later DOAC initiation (95% CI)	Net clinical benefit of early over later DOAC initiation (95% CI)	Number needed to treat
1.5	2.80 (1.74 to 3.99)	4.53 (3.23 to 5.86)	+1.73 (+0.06 to +3.40)	57.8
1.6	2.83 (1.81 to 4.05)	4.56 (3.24 to 5.91)	+1.73 (-0.08 to +3.53)	57.8
1.7	2.87 (1.81 to 4.01)	4.60 (3.40 to 5.87)	+1.73 (+0.01 to +3.56)	57.8
1.8	2.90 (1.86 to 4.11)	4.63 (3.34 to 6.03)	+1.73 (-0.09 to +3.58)	57.8
1.9	2.93 (1.85 to 4.17)	4.66 (3.38 to 6.09)	+1.73 (+0.02 to +3.48)	57.8
2.0	2.97 (1.86 to 4.29)	4.69 (3.39 to 6.12)	+1.73 (-0.15 to +3.61)	57.8
2.1	3.00 (1.91 to 4.22)	4.73 (3.37 to 6.19)	+1.73 (-0.18 to +3.65)	57.8
2.2	3.03 (1.86 to 4.32)	4.76 (3.39 to 6.21)	+1.73 (-0.24 to +3.66)	57.8
2.3	3.07 (1.87 to 4.43)	4.79 (3.43 to 6.21)	+1.73 (-0.32 to +3.70)	57.8
2.4	3.10 (1.89 to 4.41)	4.82 (3.45 to 6.41)	+1.72 (-0.33 to +3.83)	58.1
2.5	3.13 (1.95 to 4.52)	4.86 (3.47 to 6.31)	+1.72 (-0.22 to +3.71)	58.1
2.6	3.17 (1.92 to 4.55)	4.89 (3.44 to 6.35)	+1.72 (-0.31 to +3.84)	58.1
2.7	3.20 (1.90 to 4.64)	4.92 (3.52 to 6.40)	+1.72 (-0.35 to +3.75)	58.1
2.8	3.23 (1.99 to 4.65)	4.95 (3.52 to 6.52)	+1.72 (-0.46 to +3.88)	58.1
2.9	3.27 (1.92 to 4.73)	4.99 (3.56 to 6.43)	+1.72 (-0.48 to +3.73)	58.1
3.0	3.30 (1.99 to 4.91)	5.02 (3.57 to 6.55)	+1.72 (-0.56 to +3.78)	58.1
3.1	3.33 (2.01 to 4.93)	5.05 (3.57 to 6.65)	+1.72 (-0.73 to +3.97)	58.1
3.2	3.37 (2.05 to 4.98)	5.08 (3.55 to 6.72)	+1.72 (-0.70 to +3.88)	58.1
3.3	3.40 (2.08 to 5.08)	5.12 (3.65 to 6.85)	+1.72 (-0.63 to +3.98)	58.1

*calculated as [rate of recurrent ischemic stroke + (0.9 x rate of systemic embolism) + (ICH weight x rate of ICH) + (0.7 x rate of major bleeding)]. All estimates are rates of weighted events per 100 participants.

© 2025 Polymeris AA et al. *JAMA Network Open*.

eTable 2. Baseline characteristics of participants included in the ancillary NCB analysis at 90 days

Characteristic	Early DOAC initiation (N = 958)	Later DOAC initiation (N = 959)
**age**, median (IQR)	77 (70-83)	78 (70-84)
**female sex**, No. (%)	436 (45.5)	426 (44.4)
**stroke severity according to infarct size**		
**minor**	363 (37.9)	358 (37.3)
**moderate**	383 (40.0)	384 (40.0)
**major**	212 (22.1)	217 (22.6)
**NIHSS score**, median (IQR)	2.5 (1-6)	3 (1-6)
**CHA_2DS_2-VASc score**, median (IQR)	5 (4-6)	5 (4-6)
**history of**, No. (%)		
**ischemic stroke or TIA**	152/949 (16.0)	168/955 (17.6)
**systemic embolism**	18/948 (1.9)	27/953 (2.8)
**hypertension**	656/948 (69.2)	639/953 (67.1)
**myocardial infarction**	74/948 (7.8)	82/950 (8.6)
**heart failure**	59/894 (6.6)	57/895 (6.4)
**peripheral artery disease**	33/925 (3.6)	45/929 (4.8)
**diabetes mellitus**	172/948 (18.1)	150/955 (15.7)
**dyslipidemia**	419/928 (45.2)	406/935 (43.4)
**current/past smoking**	241/907 (26.6)	233/905 (25.7)
**creatinine clearance** (mL/min/1.72m^2), median (IQR)	71 (60-86)	70 (57-87)
**pre-stroke mRS score ≤ 2**, No. (%)	854/957 (89.2)	864/958 (90.2)
**acute reperfusion therapy**, No. (%)		
**intravenous thrombolysis**	204/938 (21.3)	230/939 (23.7)
**thrombectomy**	383/938 (40.0)	374/939 (38.6)
**DOAC type**, No. (%)		
**once-daily DOAC**	190/953 (19.9)	198/947 (20.9)
**twice-daily DOAC**	763/953 (80.1)	749/947 (79.1)
**DOAC dose according to the SPCs**, No. (%)		
**full dose**	781/956 (81.7)	775/953 (81.3)
**reduced dose**	175/956 (18.3)	178/953 (18.7)

NCB, Net Clinical Benefit; NIHSS, National Institutes of Health Stroke Scale at randomization; CHA_2DS_2-VASc, congestive heart failure, hypertension, age >75 years, diabetes, stroke or TIA, vascular disease, age 65 to 74 years, and sex; TIA, transient ischemic attack; mRS, modified Rankin Scale; DOAC, direct oral anticoagulant; SPCs, summary of product characteristics

© 2025 Polymeris AA et al. *JAMA Network Open*.

eTable 3. 90-Day outcomes according to treatment allocation and 30-day outcomes according to treatment allocation and infarct size

A. 90-day outcomes according to treatment allocation

**90-day outcomes**, No. (%)	**Total (N = 1917)***	**Early DOAC initiation (N = 958)**	**Later DOAC initiation (N = 959)**
**recurrent ischemic stroke**	48 (2.5)	18 (1.9)	30 (3.1)
**systemic embolism**	14 (0.7)	4 (0.4)	10 (1.0)
**major extracranial bleeding**	11 (0.6)	3 (0.3)	8 (0.8)
**symptomatic intracranial hemorrhage**	4 (0.2)	2 (0.2)	2 (0.2)

* Of 2013 participants in the full ELAN dataset, 18 (9 early in the early, 9 in the later arm) withdrew consent, 5 (3 in the early, 2 in the later arm) were lost to follow-up, and 73 participants (36 in the early, 37 in the later arm) had all-cause death as first event by 90 days.

B. 30-day outcomes according to treatment allocation and infarct size

**30-day outcomes, minor stroke**, No. (%)	**Total (N = 740)**	**Early DOAC initiation (N = 372)**	**Later DOAC initiation (N = 368)**
**recurrent ischemic stroke**	12 (1.6)	5 (1.3)	7 (1.9)
**systemic embolism**	4 (0.5)	3 (0.8)	1 (0.3)
**major extracranial bleeding**	3 (0.4)	1 (0.3)	2 (0.5)
**symptomatic intracranial hemorrhage**	0 (0.0)	0 (0.0)	0 (0.0)
**30-day outcomes, moderate stroke**, No. (%)	**Total (N = 782)**	**Early DOAC initiation (N = 389)**	**Later DOAC initiation (N = 393)**
**recurrent ischemic stroke**	19 (2.4)	7 (1.8)	12 (3.1)
**systemic embolism**	2 (0.3)	0 (0.0)	2 (0.5)
**major extracranial bleeding**	2 (0.3)	2 (0.5)	0 (0.0)
**symptomatic intracranial hemorrhage**	2 (0.3)	2 (0.5)	0 (0.0)
**30-day outcomes, major stroke**, No. (%)	**Total (N = 444)**	**Early DOAC initiation (N = 216)**	**Later DOAC initiation (N = 228)**
**recurrent ischemic stroke**	8 (1.8)	2 (0.9)	6 (2.6)
**systemic embolism**	7 (1.6)	1 (0.5)	6 (2.6)
**major extracranial bleeding**	3 (0.7)	0 (0.0)	3 (1.3)

© 2025 Polymeris AA et al. *JAMA Network Open*.

**symptomatic intracranial hemorrhage**	2 (0.5)	0 (0.0)	2 (0.9)

© 2025 Polymeris AA et al. *JAMA Network Open*.

eTable 4. Net clinical benefit of early over later DOAC initiation at 90 days

ICH weight	weighted rate of events* in participants with early DOAC initiation (95% CI)	weighted rate of events* in participants with later DOAC initiation (95% CI)	Net clinical benefit of early over later DOAC initiation (95% CI)	Number needed to treat
1.5	3.20 (2.11 to 4.38)	5.35 (3.97 to 6.70)	+2.16 (+0.30 to +3.87)	46.3
1.6	3.23 (2.16 to 4.55)	5.38 (3.96 to 6.83)	+2.15 (+0.05 to +3.99)	46.5
1.7	3.26 (2.20 to 4.44)	5.42 (4.05 to 6.76)	+2.15 (+0.37 to +4.06)	46.5
1.8	3.30 (2.15 to 4.59)	5.45 (3.98 to 6.90)	+2.15 (+0.20 to +4.12)	46.5
1.9	3.33 (2.17 to 4.63)	5.48 (4.16 to 6.96)	+2.15 (+0.32 to +4.03)	46.5
2.0	3.36 (2.21 to 4.74)	5.51 (4.06 to 7.05)	+2.15 (+0.09 to +4.21)	46.5
2.1	3.40 (2.23 to 4.77)	5.55 (4.12 to 7.02)	+2.15 (+0.06 to +4.17)	46.5
2.2	3.43 (2.19 to 4.74)	5.58 (4.12 to 7.10)	+2.15 (+0.02 to +4.30)	46.5
2.3	3.46 (2.23 to 4.86)	5.61 (4.16 to 7.11)	+2.15 (+0.02 to +4.15)	46.5
2.4	3.50 (2.25 to 4.91)	5.64 (4.24 to 7.36)	+2.15 (+0.13 to +4.28)	46.5
2.5	3.53 (2.31 to 4.97)	5.68 (4.27 to 7.20)	+2.15 (+0.08 to +4.27)	46.5
2.6	3.56 (2.27 to 4.90)	5.71 (4.16 to 7.31)	+2.15 (+0.14 to +4.21)	46.5
2.7	3.60 (2.27 to 5.09)	5.74 (4.20 to 7.36)	+2.14 (-0.06 to +4.32)	46.7
2.8	3.63 (2.33 to 5.08)	5.77 (4.24 to 7.42)	+2.14 (-0.07 to +4.48)	46.7
2.9	3.66 (2.30 to 5.16)	5.81 (4.31 to 7.37)	+2.14 (-0.07 to +4.34)	46.7
3.0	3.70 (2.33 to 5.35)	5.84 (4.23 to 7.48)	+2.14 (-0.33 to +4.38)	46.7
3.1	3.73 (2.29 to 5.41)	5.87 (4.28 to 7.61)	+2.14 (-0.34 to +4.42)	46.7
3.2	3.76 (2.41 to 5.49)	5.90 (4.33 to 7.61)	+2.14 (-0.37 to +4.42)	46.7
3.3	3.80 (2.47 to 5.61)	5.93 (4.32 to 7.74)	+2.14 (-0.26 to +4.41)	46.7

*calculated as [rate of recurrent ischemic stroke + (0.9 x rate of systemic embolism) + (ICH weight x rate of ICH) + (0.7 x rate of major bleeding)]. All estimates are rates of weighted events per 100 participants.

© 2025 Polymeris AA et al. *JAMA Network Open*.

eTable 5. Net clinical benefit of early over later DOAC initiation at 30 days in subgroups according to infarct size

ICH weight	Minor stroke			Moderate stroke			Major stroke		
	weighted rate of events* in early treatment (95% CI)	weighted rate of events* in later treatment (95% CI)	Net clinical benefit of early over later treatment (95% CI)	weighted rate of events* in early treatment (95% CI)	weighted rate of events* in later treatment (95% CI)	Net clinical benefit of early over later treatment (95% CI)	weighted rate of events* in early treatment (95% CI)	weighted rate of events* in later treatment (95% CI)	Net clinical benefit of early over later treatment (95% CI)
1.5	3.34 (1.95 to 5.00)	3.56 (2.15 to 5.22)	+0.22 (-2.21 to +2.43)	4.04 (2.13 to 6.22)	4.44 (2.80 to 6.44)	+0.40 (-2.36 to +3.14)	2.65 (1.22 to 4.71)	9.34 (5.84 to 13.03)	+6.69 (+2.68 to +10.68)
1.6	3.37 (2.00 to 4.97)	3.59 (2.18 to 5.21)	+0.22 (-1.93 to +2.43)	4.12 (2.25 to 6.57)	4.45 (2.67 to 6.36)	+0.33 (-2.79 to +3.34)	2.68 (1.29 to 4.56)	9.49 (5.80 to 13.88)	+6.81 (+2.67 to +11.44)
1.7	3.40 (2.04 to 4.96)	3.61 (2.11 to 5.39)	+0.21 (-2.05 to +2.64)	4.21 (2.29 to 6.54)	4.47 (2.71 to 6.37)	+0.26 (-2.92 to +3.03)	2.71 (1.26 to 4.69)	9.63 (5.68 to 13.87)	+6.92 (+2.51 to +11.27)
1.8	3.42 (1.96 to 5.04)	3.63 (2.11 to 5.38)	+0.21 (-2.28 to +2.76)	4.29 (2.35 to 6.63)	4.49 (2.79 to 6.50)	+0.19 (-2.68 to +3.18)	2.74 (1.28 to 4.68)	9.77 (6.10 to 14.04)	+7.03 (+2.51 to +11.94)
1.9	3.45 (2.12 to 5.08)	3.66 (2.17 to 5.48)	+0.21 (-2.12 to +2.63)	4.38 (2.43 to 6.72)	4.51 (2.80 to 6.40)	+0.13 (-2.91 to +3.05)	2.77 (1.36 to 4.65)	9.91 (6.09 to 14.47)	+7.14 (+2.40 to +11.84)
2.0	3.48 (2.07 to 5.15)	3.68 (2.15 to 5.27)	+0.21 (-2.15 to +2.43)	4.46 (2.29 to 7.03)	4.52 (2.83 to 6.48)	+0.06 (-3.09 to +3.27)	2.80 (1.37 to 4.63)	10.05 (6.22 to 14.17)	+7.25 (+2.61 to +11.74)
2.1	3.50 (1.99 to 5.12)	3.71 (2.25 to 5.30)	+0.20 (-2.13 to +2.57)	4.55 (2.31 to 7.20)	4.54 (2.87 to 6.60)	-0.01 (-3.17 to +3.13)	2.84 (1.40 to 4.66)	10.19 (6.17 to 14.51)	+7.36 (+2.40 to +12.03)
2.2	3.53 (2.01 to 5.12)	3.73 (2.24 to 5.45)	+0.20 (-2.23 to +2.40)	4.63 (2.39 to 7.21)	4.56 (2.83 to 6.60)	-0.08 (-3.42 to +3.17)	2.87 (1.40 to 4.66)	10.33 (6.36 to 15.03)	+7.47 (+2.93 to +12.35)
2.3	3.56 (2.16 to 5.30)	3.76 (2.26 to 5.50)	+0.20 (-2.07 to +2.63)	4.72 (2.56 to 7.49)	4.57 (2.80 to 6.66)	-0.15 (-3.41 to +2.82)	2.90 (1.49 to 4.82)	10.48 (6.63 to 15.08)	+7.58 (+3.12 to +12.12)
2.4	3.59 (2.19 to 5.23)	3.78 (2.22 to 5.48)	+0.20 (-2.02 to +2.44)	4.80 (2.34 to 7.71)	4.59 (2.91 to 6.57)	-0.21 (-3.60 to +3.35)	2.93 (1.44 to 4.80)	10.62 (6.31 to 15.43)	+7.69 (+2.71 to +12.99)
2.5	3.61 (2.08 to 5.19)	3.81 (2.32 to 5.60)	+0.19 (-2.06 to +2.54)	4.89 (2.46 to 7.82)	4.61 (2.91 to 6.51)	-0.28 (-3.71 to +3.01)	2.96 (1.52 to 4.99)	10.76 (6.29 to 15.74)	+7.80 (+2.56 to +13.22)
2.6	3.64 (2.25 to 5.40)	3.83 (2.37 to 5.49)	+0.19 (-2.25 to +2.52)	4.97 (2.38 to 7.73)	4.62 (2.98 to 6.69)	-0.35 (-3.47 to +3.19)	2.99 (1.55 to 5.12)	10.90 (6.23 to 15.72)	+7.91 (+2.42 to +13.22)
2.7	3.67 (2.30 to 5.28)	3.86 (2.39 to 5.39)	+0.19 (-2.09 to +2.43)	5.06 (2.64 to 8.09)	4.64 (2.90 to 6.74)	-0.42 (-4.10 to +2.89)	3.02 (1.59 to 4.89)	11.04 (6.65 to 15.80)	+8.02 (+3.25 to +13.30)
2.8	3.69 (2.20 to 5.43)	3.88 (2.38 to 5.59)	+0.19 (-2.03 to +2.65)	5.14 (2.60 to 8.34)	4.66 (2.93 to 6.71)	-0.48 (-4.26 to +2.97)	3.05 (1.60 to 5.02)	11.18 (6.72 to 16.80)	+8.13 (+2.93 to +14.04)
2.9	3.72 (2.33 to 5.47)	3.91 (2.39 to 5.54)	+0.19 (-2.37 to +2.57)	5.23 (2.57 to 8.26)	4.68 (2.99 to 6.63)	-0.55 (-4.36 to +2.98)	3.08 (1.56 to 5.10)	11.33 (6.65 to 16.78)	+8.24 (+2.86 to +14.28)
3.0	3.75 (2.32 to 5.49)	3.93 (2.37 to 5.51)	+0.18 (-2.35 to +2.36)	5.31 (2.62 to 8.44)	4.69 (2.88 to 6.84)	-0.62 (-4.34 to +3.00)	3.11 (1.68 to 5.00)	11.47 (6.85 to 17.19)	+8.35 (+2.90 to +14.56)
3.1	3.77 (2.39 to 5.37)	3.96 (2.46 to 5.74)	+0.18 (-2.20 to +2.65)	5.40 (2.78 to 9.18)	4.71 (2.99 to 6.72)	-0.69 (-4.83 to +2.81)	3.14 (1.79 to 5.18)	11.61 (7.11 to 17.02)	+8.46 (+3.47 to +14.20)
3.2	3.80 (2.41 to 5.41)	3.98 (2.53 to 5.76)	+0.18 (-1.97 to +2.77)	5.48 (2.63 to 9.13)	4.73 (3.03 to 6.73)	-0.76 (-4.55 to +2.93)	3.18 (1.75 to 5.17)	11.75 (7.00 to 17.53)	+8.57 (+3.23 to +14.65)
3.3	3.83 (2.38 to 5.52)	4.00 (2.55 to 5.67)	+0.18 (-2.22 to +2.49)	5.57 (2.71 to 9.45)	4.74 (3.04 to 6.67)	-0.82 (-5.07 to +2.81)	3.21 (1.72 to 5.30)	11.89 (7.04 to 17.31)	+8.69 (+3.01 to +14.74)

*calculated as [rate of recurrent ischemic stroke + (0.9 x rate of systemic embolism) + (ICH weight x rate of ICH) + (0.7 x rate of major bleeding)]. All estimates are rates of weighted events per 100 participants.

© 2025 Polymeris AA et al. *JAMA Network Open*.

eTable 6. Net clinical benefit of early over later DOAC initiation at 30 days including non-major bleeding with a weight of 0.1 and 0.5

ICH weights	Non-major bleeding weight of 0.1			Non-major bleeding weight of 0.5		
	weighted rate of events* in early treatment (95% CI)	weighted rate of events* in later treatment (95% CI)	Net clinical benefit of early over later treatment (95% CI)	weighted rate of events* in early treatment (95% CI)	weighted rate of events* in later treatment (95% CI)	Net clinical benefit of early over later treatment (95% CI)
1.5	3.11 (2.03 to 4.30)	4.81 (3.53 to 6.15)	+1.70 (+0.03 to +3.36)	4.36 (3.12 to 5.68)	5.94 (4.54 to 7.34)	+1.59 (-0.38 to +3.51)
1.6	3.14 (2.09 to 4.35)	4.85 (3.53 to 6.22)	+1.70 (-0.11 to +3.51)	4.39 (3.19 to 5.72)	5.97 (4.49 to 7.47)	+1.59 (-0.46 to +3.53)
1.7	3.18 (2.11 to 4.39)	4.88 (3.63 to 6.16)	+1.70 (-0.07 to +3.50)	4.42 (3.26 to 5.79)	6.01 (4.65 to 7.38)	+1.58 (-0.41 to +3.55)
1.8	3.21 (2.15 to 4.44)	4.91 (3.60 to 6.33)	+1.70 (-0.16 to +3.56)	4.46 (3.17 to 5.81)	6.04 (4.60 to 7.53)	+1.58 (-0.53 to +3.67)
1.9	3.24 (2.16 to 4.53)	4.94 (3.66 to 6.38)	+1.70 (-0.07 to +3.51)	4.49 (3.30 to 5.93)	6.07 (4.71 to 7.67)	+1.58 (-0.48 to +3.50)
2.0	3.28 (2.18 to 4.58)	4.98 (3.67 to 6.36)	+1.70 (-0.22 to +3.58)	4.52 (3.32 to 5.89)	6.10 (4.70 to 7.58)	+1.58 (-0.45 to +3.58)
2.1	3.31 (2.17 to 4.54)	5.01 (3.65 to 6.53)	+1.70 (-0.23 to +3.55)	4.56 (3.24 to 5.91)	6.14 (4.70 to 7.73)	+1.58 (-0.54 to +3.60)
2.2	3.34 (2.15 to 4.65)	5.04 (3.65 to 6.48)	+1.70 (-0.26 to +3.62)	4.59 (3.28 to 6.05)	6.17 (4.70 to 7.69)	+1.58 (-0.48 to +3.66)
2.3	3.38 (2.17 to 4.80)	5.07 (3.73 to 6.50)	+1.70 (-0.38 to +3.71)	4.62 (3.37 to 6.19)	6.20 (4.70 to 7.71)	+1.58 (-0.76 to +3.71)
2.4	3.41 (2.18 to 4.74)	5.11 (3.74 to 6.68)	+1.70 (-0.39 to +3.81)	4.66 (3.33 to 6.07)	6.23 (4.87 to 7.93)	+1.58 (-0.59 to +3.91)
2.5	3.44 (2.28 to 4.85)	5.14 (3.76 to 6.60)	+1.69 (-0.23 to +3.72)	4.69 (3.42 to 6.22)	6.27 (4.85 to 7.81)	+1.58 (-0.65 to +3.67)
2.6	3.48 (2.19 to 4.84)	5.17 (3.70 to 6.65)	+1.69 (-0.35 to +3.80)	4.72 (3.24 to 6.17)	6.30 (4.76 to 7.89)	+1.58 (-0.53 to +3.91)
2.7	3.51 (2.23 to 4.96)	5.20 (3.79 to 6.65)	+1.69 (-0.47 to +3.72)	4.76 (3.36 to 6.21)	6.33 (4.78 to 7.85)	+1.58 (-0.66 to +3.80)
2.8	3.54 (2.26 to 4.97)	5.24 (3.78 to 6.87)	+1.69 (-0.49 to +3.88)	4.79 (3.34 to 6.43)	6.36 (4.85 to 8.04)	+1.57 (-0.72 to +3.93)
2.9	3.58 (2.23 to 5.04)	5.27 (3.83 to 6.78)	+1.69 (-0.51 to +3.72)	4.82 (3.35 to 6.39)	6.40 (4.90 to 8.01)	+1.57 (-0.72 to +3.82)
3.0	3.61 (2.28 to 5.23)	5.30 (3.86 to 6.85)	+1.69 (-0.61 to +3.80)	4.86 (3.38 to 6.61)	6.43 (4.86 to 8.12)	+1.57 (-0.86 to +3.82)
3.1	3.64 (2.32 to 5.26)	5.33 (3.86 to 6.92)	+1.69 (-0.75 to +3.95)	4.89 (3.43 to 6.69)	6.46 (4.86 to 8.17)	+1.57 (-1.00 to +3.94)
3.2	3.68 (2.35 to 5.33)	5.37 (3.84 to 7.02)	+1.69 (-0.72 to +3.82)	4.92 (3.39 to 6.68)	6.49 (4.90 to 8.25)	+1.57 (-0.97 to +3.93)
3.3	3.71 (2.37 to 5.39)	5.40 (3.90 to 7.12)	+1.69 (-0.70 to +4.00)	4.96 (3.49 to 6.72)	6.53 (4.94 to 8.25)	+1.57 (-0.97 to +4.02)

*calculated as [rate of recurrent ischemic stroke + (0.9 x rate of systemic embolism) + (ICH weight x rate of ICH) + (0.7 x rate of major bleeding) + (non-major bleeding weight x rate of non-major bleeding)]. All estimates are rates of weighted events per 100 participants.

© 2025 Polymeris AA et al. *JAMA Network Open*.

eFigure 1. Net clinical benefit of early over later DOAC initiation at 30 days in weighted events possibly prevented per 100 participants (solid line) with 95% confidence intervals (grey shaded area) in subgroups according to infarct size.

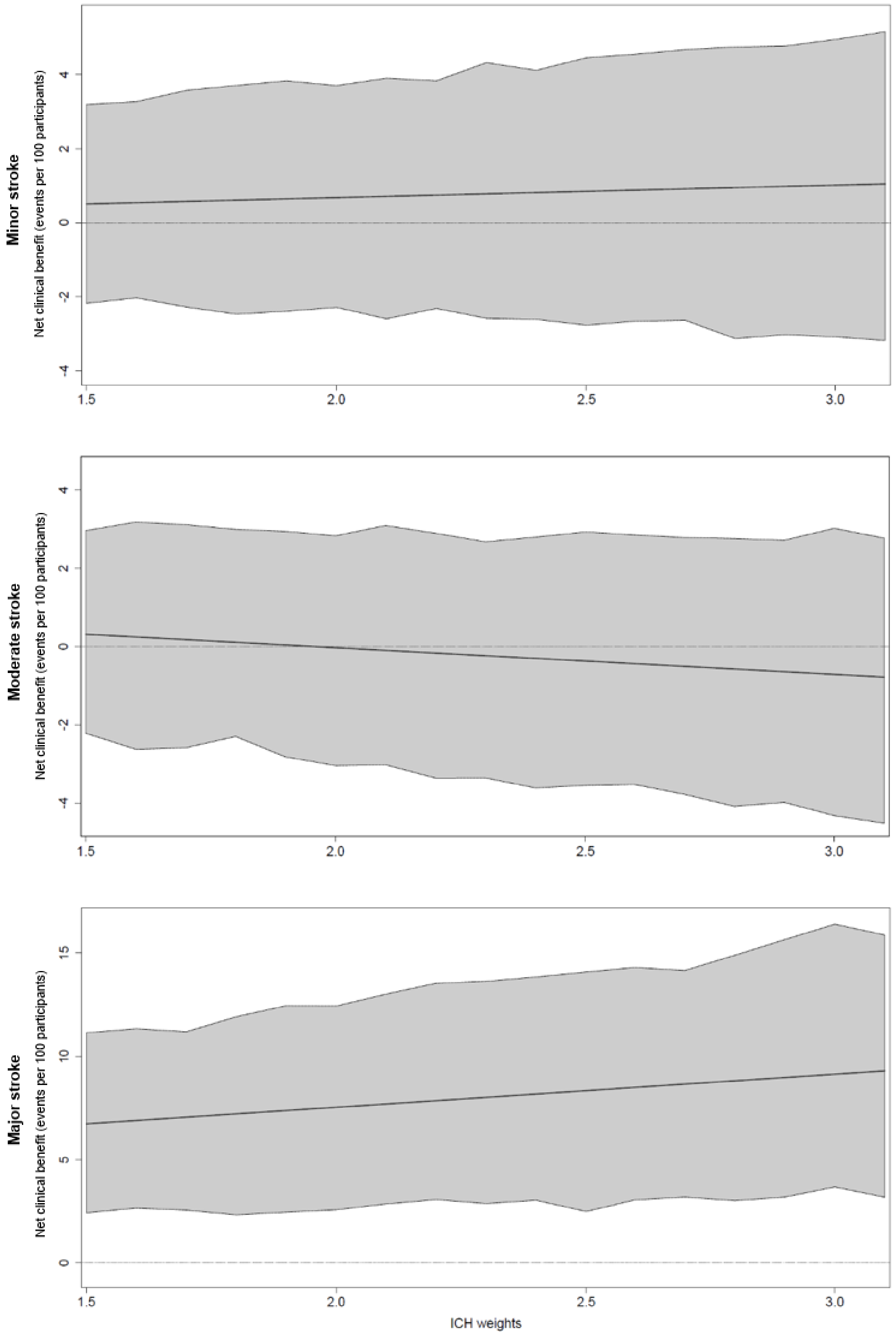

© 2025 Polymeris AA et al. *JAMA Network Open*.

eFigure 2. Net clinical benefit of early over later DOAC initiation at 30 days in weighted events possibly prevented per 100 participants (solid line) with 95% confidence intervals (grey shaded area), including non-major bleeding events with a weight of 0.1 and 0.5

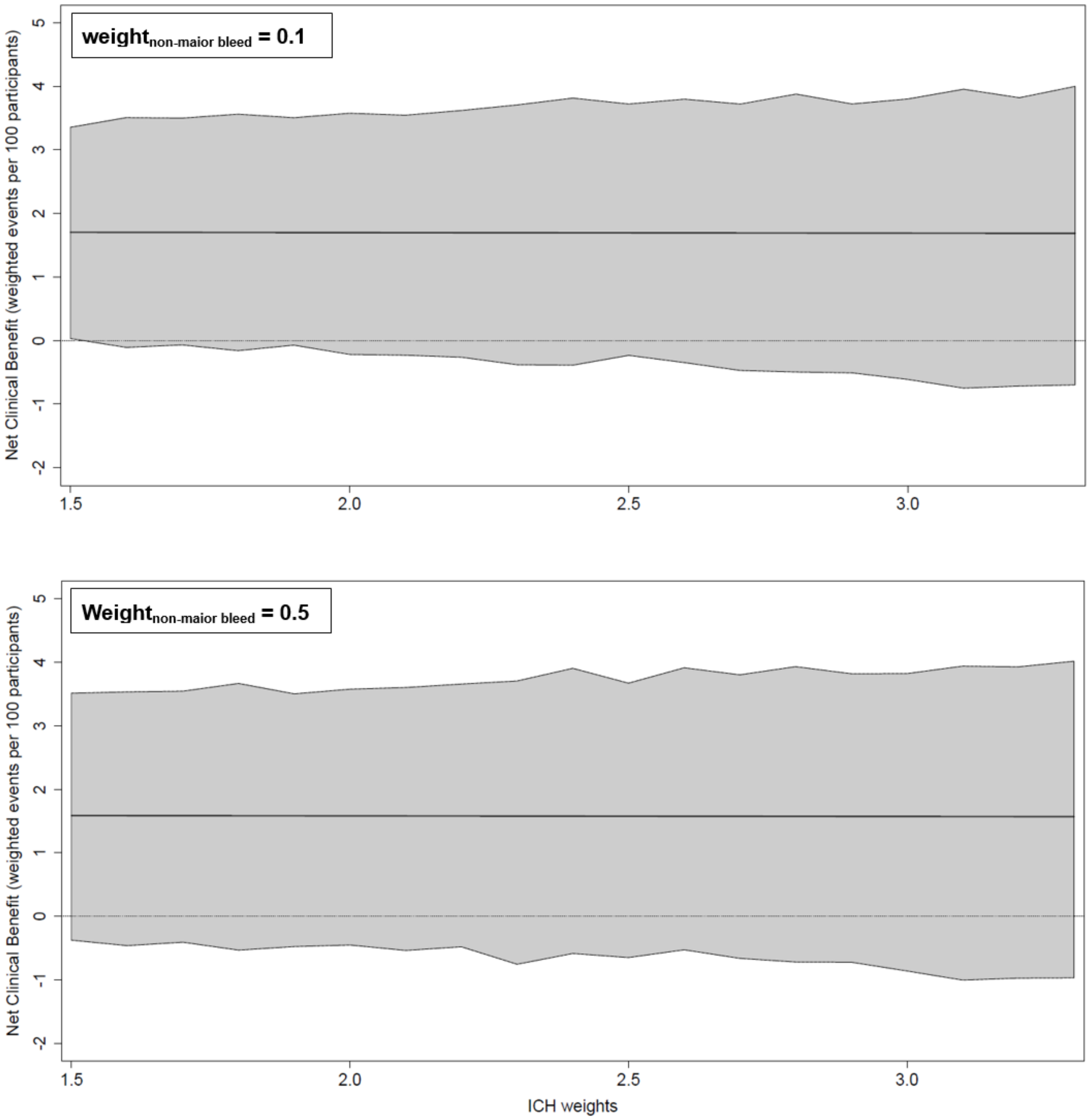

© 2025 Polymeris AA et al. *JAMA Network Open*.
