# Supplementary material for: Net Benefit of Early Anticoagulation for Stroke With Atrial Fibrillation: Post Hoc Analysis of the ELAN Randomized Clinical Trial
Source: JAMA Netw Open. 2025 Jan 28;8(1):e2456307. doi: 10.1001/jamanetworkopen.2024.56307 (PMC11775740; doi:10.1001/jamanetworkopen.2024.56307)
Supplement: Supplement 3. — Nonauthor Collaborators. The ELAN Investigators [file jamanetwopen-e2456307-s003.pdf]

Supplemental Online Content: Nonauthor Collaborators
*First name, last name, and suffix (if applicable) are required and will appear in PubMed.

*Group Name(s): ELAN Investigators							
*First Name and Middle Initial(s)	*Last Name	*Suffix (eg, Jr, III)	Academic Degrees	Institution	Location (city, state/province, country)	Role or Contribution, eg, chair, principal investigator	Group (if more than 1 Group listed in the byline) and/or Subgroup (eg, Steering Committee)
Adedolapo Kamaldee	Adeyemi		MD	Department of Neurology & Stroke, Tübingen University	Tübingen, Germany	Sub-investigator	
Adhiyaman	Vedamurthy		MD	Glan Clwyd Hospital, Betsi Cadwaladr University Local Health Board, Rhyl	Rhyl, United Kingdom	Principle investigator	
Adrian	Scutelnic		MD	Department of Neurology, University Hospital Bern, University of Bern	Bern, Switzerland	Sub-investigator	
Akiyama	Hisanao		MD, PhD	Department of Neurology, St. Marianna University School of Medicine	Kawasaki, Japan	Principle investigator	
Alastair	Wilson		PhD	School of Cardiovascular and Metabolic Health, University of Glasgow	Glasgow, United Kingdom	National UK trial Coordinator	
Alexander Andrea Tarnutzer			MD	Department of Neurology, Cantonal Hospital of Baden<br>Faculty of Medicine, University of Zurich	Baden, Switzerland<br>Zurich, Switzerland	Principle investigator	
Alexander	Pichler		MD, PhD	Department of Neurology, Medical University of Graz	Graz, Austria	Sub-investigator	
Alexander	Salerno		MD	Department of Neurology, University Hospital Lausanne, University of Lausanne	Lausanne, Switzerland	Sub-investigator	
Alexander	Vanhoorne		MD	Department of Neurology, AZ Groeninge Kortrijk	Kortrijk, Belgium	Sub-investigator	
Ami	Wilkinson		BSc	Stroke Department, University Hospital of North Durham	Durham, United Kingdom	Study nurse / trial coordinator	
Ana	Paiva Nunes		MD	Stroke Unit, Lisbon Central University Hospital	Lisbon, Portugal	Principle investigator	
Anastasia	Adamou		MD	Department of Internal Medicine, School of Health Sciences, University of Thessaly	Larissa, Greece	Sub-investigator	
André	Peeters		MD	University Hospital Saint-Luc Brussels	Brussels, Belgium	Principle investigator	
Andrea M.	Humm		MD	Department of Internal Medicine, Division of Neurology, HFR Fribourg –	Fribourg, Switzerland	Principle investigator	
Andrea	Zini		MD, FESO	IRCCS Istituto delle Scienze Neurologiche di Bologna, Department of	Bologna, Italy	Principle investigator	
Aneesh	Dhasan		PhD	Sree Chitra Tirunal Institute for Medical Sciences and Technology	Kerala, India	Local trial Coordinator	
Angelika	Alonso		MD	Department of Neurology, Medical Faculty Mannheim, University of Heidelberg	Heidelberg, Germany	Principle investigator	
Anna	Fischer		MD	Department Neurology, University Clinic St. Pölten, Karl Landsteiner Private University for Health Sciences	St. Pölten, Austria	Sub-investigator	
Anna Maija	Saukkonen		MD	Department of Neurology, North Karelia Central Hospital,	Joensuu, Finland	Sub-investigator	
Anna	Müller		RN	Department of Neurology, Cantonal Hospital St. Gallen	St. Gallen, Switzerland	Study nurse / trial coordinator	
Anne	Berberich		MD	Department of Neurology, Heidelberg University Hospital	Heidelberg, Germany	Sub-investigator	
Anne	Falcou		MD, PhD	Department of Emergency, Policlinico Umberto I,	Rome, Italy	Sub-investigator	
Annemie	Devroye		BSc	Department of Neurology, University Hospitals Leuven	Leuven, Belgium	Nationale Belgium Coordinator	
Arne	Hostens		MD	Onze-Lieve-Vrouw Ziekenhuis	Aalst, Belgium	Sub-investigator	
Arsany	Hakim		MD	University Institute of Diagnostic and Interventional Neuroradiology, Inselspital Bern	Bern, Switzerland	Imaging Core Lab	
Arthur	Liesz		MD	Institute for Stroke and Dementia Research, University Hospital, Ludwig Maximilians University of Munich	Munich, Germany	Sub-investigator	

*First Name and Middle Initial(s)	*Last Name	*Suffix (eg, Jr, III)	Academic Degrees	Institution	Location (city, state/province, country)	Role or Contribution, eg, chair, principal investigator	Group (if more than 1 Group listed in the byline) and/or Subgroup (eg, Steering Committee)
Arunkumar	Annamalai		MBBS	North Tees and Hartlepool NHS Foundation Trust	Stockton on Tees, United Kingdom	Principle investigator	
Arvind Vijaysharan	Sharma		MD, DM	Zydus Hospitals & Healthcare Research	Thaltej Ahmedabad, India	Principle investigator	
Asterios	Paliantonis		MD	Stroke Center, Hirslanden Clinic	Zurich, Switzerland	Sub-investigator	
Aumugam	Nallasivan		MRCP	Countess of Chester Hospital NHS Foundation Trust	Chester, Cheshire, United Kingdom	Principle investigator	
Azmil	Abdul-Rahim		MD	School of Cardiovascular and Metabolic Health, University of Glasgow	Glasgow, United Kingdom	Sub-investigator	
Beata	Rezny-Kasprzak		MD	University Institute of Diagnostic and Interventional Neuroradiology, Inselspital, Bern University Hospital, University of Bern	Bern, Switzerland	Sub-investigator	
Ben	Grimshaw		MBChB	Southmead Hospital, North Bristol NHS Trust	Bristol, United Kingdom	Principle investigator	
Bernd	Kallmünzer		MD	Department of Neurology, Universitätsklinikum Erlangen, Friedrich-Alexander University Erlangen-Nürnberg	Erlangen, Germany	Principle investigator	
Biljana	Rodic		MD	Department of Neurology, Cantonal Hospital Winterthur	Winterthur, Switzerland	Sub-investigator	
Brian	Clarke		MD	Neurology Department, St George's University Hospital	London, United Kingdom	Sub-investigator	
Brian	Menezes		MBBS, MRCP	Stroke Department, Wirral University Hospital, Wirral NHS Foundation Trust	Wirral, United Kingdom	Principle investigator	
Bruno J.	Weder		MD	Support Centre for Advanced Neuroimaging, Institute for Diagnostic and Interventional Neuroradiology, University Hospital Bern	Bern, Switzerland	CEC member	
Carla	Ciobanu		MD	Department of Neurology, Comprehensive Stroke Unit, CHC MontLégia Hospital	Liège, Belgium	Sub-investigator	
Carlo W.	Cereda		MD	Stroke Center EOC, Neurocenter of Southern Switzerland, Ospedale Civico	Lugano, Switzerland	Principle investigator	
Caroline	Loos		MD, PhD	NeuroVascular Center, Stroke Unit Antwerp, Department of Neurology, Antwerp University Hospital, Translational Neurosciences, Faculty of Medicine and Health Sciences, University of Antwerp	Antwerp, Belgium	Study nurse / trial coordinator	
Caterina	Kulyk		MD	Department of Neurology 2, Kepler University Hospital, Johannes Kepler University	Linz, Austria	Principle investigator	
Catia	Gonçalves Martins			Department of Neurology, Valais Hospital	Sion, Switzerland	Study nurse / trial coordinator	
Cecilia	Ferrari		MBA	Department of Neurology, University Hospital Bern, University of Bern	Bern, Switzerland	Sponsor - Trial Team	
Christian	Fung		MD	Department of Neurosurgery, Medical Center, University of Freiburg	Freiburg, Germany	CEC member	
Christina	Caporale		MD	Stroke Unit, Cantonal Hospital Graubünden	Graubünden, Switzerland	Sub-investigator	
Christine	McAlpine		MBChB	Acute Stroke Unit, Glasgow Royal Infirmary	Glasgow, United Kingdom	Sub-investigator	
Christoph	Globas		MD	Department of Neurology, University Hospital Zurich	Zurich, Switzerland	Principle investigator	
Christoph	Gumbinger		MD	Department of Neurology, Heidelberg University Hospital	Heidelberg, Germany	Principle investigator	
Christophe	Bonvin		MD, MSc	Department of Neurology, Valais Hospital	Sion, Switzerland	Principle investigator	
Christos	Krogias		MD, FESO	Department of Neurology, Ruhr University Bochum, St. Josef-Hospital	Bochum, Germany	Principle investigator	

*First Name and Middle Initial(s)	*Last Name	*Suffix (eg, Jr, III)	Academic Degrees	Institution	Location (city, state/province, country)	Role or Contribution, eg, chair, principal investigator	Group (if more than 1 Group listed in the byline) and/or Subgroup (eg, Steering Committee)
Clare	Whyte		BSc	Clinical Research Centre, Ninewells Hospital	Dundee, United Kingdom	Study nurse / trial coordinator	
Claudio	Bassetti		MD	Department of Neurology, University Hospital Bern, University of Bern	Bern, Switzerland	Sponsor - Trial Team	
Dan	Ryan		MD, PhD	Tallaght University Hospital	Dublin, Ireland	Principle investigator	
Daniel	Charissé		MD	Department of Neurology, Goethe-University Hospital Frankfurt	Frankfurt, Germany	Sub-investigator	
Daniel	Richter		MD	Department of Neurology, Ruhr University Bochum, St. Josef-Hospital	Bochum, Germany	Sub-investigator	
Daniel	Schrammel		MD	Department of Neurology, Hospital of the Brothers of St. John of God Eisenstadt	Eisenstadt, Austria	Sub-investigator	
Daria	Giudici		MD	Internal, Vascular and Emergency Medicine – Stroke Unit, Santa Maria della Misericordia Hospital, University of Perugia	Perugia, Italy	Sub-investigator	
Darius G.	Nabavi		MD	Department of Neurology, Vivantes Hospital Neukölln	Berlin, Germany	Principle investigator	
David	Bradley		PhD	St James's Hospital	Dublin, Ireland	Principle investigator	
David	Orion		MD	Stroke Clinic, Chaim Sheba Medical Centre, Ramat Gan	Tel Aviv, Israel	Principle investigator	
David J.	Seiffge		MD	Department of Neurology, University Hospital Bern, University of Bern	Bern, Switzerland	Sub-investigator	
David	Werring		MD	Stroke Research Centre, Department of Brain Repair and Rehabilitation, UCL Queen Square Institute of Neurology, University College London	London, United Kingdom	Associate	
Davide	Strambo		MD	Department of Neurology, Lausanne University Hospital and University of Lausanne	Lausanne, Switzerland	Sub-investigator	
Derek	Esson		BSc	University Hospital Monklands, Airdrie	Lanarkshire, United Kingdom	Study nurse / trial coordinator	
Dheeraj	Khurana		MD, DM	Department of Neurology, Postgraduate Institute of Medical Education and Research	Chandigarh, India	Principle investigator	
Diana	Melancia		MD	Stroke Unit, Lisbon Central University Hospital	Lisbon, Portugal	Sub-investigator	
Dimitre	Staykov		MD	Department of Neurology, Hospital of the Brothers of St. John of God Eisenstadt	Eisenstadt, Austria	Principle investigator	
Dimitri	Hemelsoet		MD	Department of Neurology, Ghent University Hospital	Ghent, Belgium	Principle investigator	
Dominik	Michalski		MD	Department of Neurology, University of Leipzig	Leipzig, Germany	Principle investigator	
Eckhard	Schlemm		MD, PhD	Department of Neurology, University Medical Centre Hamburg-Eppendorf	Hamburg, Germany	Sub-investigator	
Efstathia	Karagkiozi		RN, MSc	Department of Internal Medicine, School of Health Sciences, University of Thessaly	Larissa, Greece	Study nurse / trial coordinator	
Elisabeth	Olbert		MD	Department of Neurology, University Hospital Tulln	Tulln an der Donau, Austria	Sub-investigator	
Espen	Saxhaug Kristoffersen		MD, PhD	Department of Neurology, Akershus University Hospital<br>Department of General Practice, University of Oslo	Lørenskog, Norway<br>Oslo, Norway	Principle investigator	
Evelyn	Marcelis		BSc	Department of Neurology, University Hospitals Leuven	Leuven, Belgium	Study nurse / trial coordinator	
Fiona	Wright		MBChB	Acute Stroke Unit, Glasgow Royal Infirmary	Glasgow, United Kingdom	Principle investigator	

*First name, last name, and suffix (if applicable) are required and will appear in PubMed.

*First Name and Middle Initial(s)	*Last Name	*Suffix (eg, Jr, III)	Academic Degrees	Institution	Location (city, state/province, country)	Role or Contribution, eg, chair, principal investigator	Group (if more than 1 Group listed in the byline) and/or Subgroup (eg, Steering Committee)
François	Delvoye		MD	Department of Neurology, Comprehensive Stroke Unit, CHC MontLégia Hospital	Liège, Belgium	Sub-investigator	
Friedrich	Medlin		MD	Department of Internal Medicine, Division of Neurology, HFR Fribourg – Cantonal Hospital	Fribourg, Switzerland	Sub-investigator	
Fukano	Takayuki		MD	Department of Neurology, St. Marianna University School of Medicine	Kawasaki, Japan	Sub-investigator	
Gaia	Sirimarco		MD, PhD	Department of Neurology, Lausanne University Hospital and University of Lausanne	Lausanne, Switzerland	Sub-investigator	
Gek	Shim		MD	Stroke Department, University Hospital of North Durham	Durham, United Kingdom	Principle investigator	
Gemma Marie	Smith		MBBS	Stroke Department, University Hospital of North Durham	Durham, United Kingdom	Sub-investigator	
Georg	Royl		MD	Department of Neurology, Neurovascular Center, University of Lübeck	Lübeck, Germany	Principle investigator	
George	Pope		MD	University Hospital Waterford	Waterford, Ireland	Sub-investigator	
Georgia	Salanti		PhD	Institute of Social and Preventive Medicine, University of Bern	Bern, Switzerland	Statistician	
Gerli	Sibolt		MD, PhD	Department of Neurology, Helsinki University Hospital and University of Helsinki	Helsinki, Finland	Sub-investigator	
German	Guzman-Gutierrez		MD	Grampian University Hospitals NHS Trust	Aberdeen, United Kingdom	Sub-investigator	
Gian Marco	De Marchis		MD, MSc	University Hospital Basel and University of Basel	Basel, Switzerland	Principle investigator	
Giovanni	Bianco		MD	Stroke Center EOC, Neurocenter of Southern Switzerland, Ospedale Civico	Lugano, Switzerland	Sub-investigator	
Harvey	Dymond		RN	Weston General Hospital, Weston-Super-Mare	Somerset, United Kingdom	Local trial Coordinator	
Hege	Ihle-Hansen		MD, PhD	Department of Neurology, Oslo University Hospital	Oslo, Norway	Sub-investigator	
Helen	Thomas			Glan Clwyd Hospital, Betsi Cadwaladr University Local Health Board	Rhyl, United Kingdom	Study nurse / trial coordinator	
Henning R.	Stetefeld		MD	Department of Neurology, Faculty of Medicine and University Hospital Cologne, University of Cologne	Cologne, Germany	Sub-investigator	
Himanshu	Koundal		MSc	Department of Neurology, All India Institute of Medical Sciences	New Delhi, India	Study nurse / trial coordinator	
Honig	Asaf		MD	Hadassah-Hebrew University Medical Centre	Jerusalem, Israel	Sub-investigator	
Ijaz	Anwar		MBBS	North Tees and Hartlepool NHS Foundation Trust	Stockton on Tees, United Kingdom	Sub-investigator	
Ilaria Leone	De Magistris		MD	Stroke Unit – Internal, Vascular and Emergency Medicine, Santa Maria della Misericordia Hospital University of Perugia	Perugia, Italy	Sub-investigator	
Imelda	Noone		MSc	St Vincent’s University Hospital	Dublin, Ireland	Study nurse / trial coordinator	
Ingrid	Olave Bersas		MD	Aalesund Hospital, Helse More og Romsdal Health Trust	Aalesund, Norway	Sub-investigator	
Ioan-Paul	Muresan		MD	Division of Neurology, Neuchatel Hospital Network	Neuchatel, Switzerland	Principle investigator	
Isabelle	Vanpanteghem			Department of Neurology, AZ Groeninge Kortrijk	Kortrijk, Belgium	Study nurse / trial coordinator	
Jan	Gralla		MD	Department of Neurology, University Hospital Bern, University of Bern	Bern, Switzerland	Sponsor - Trial Team	

Supplemental Online Content: Nonauthor Collaborators
*First name, last name, and suffix (if applicable) are required and will appear in PubMed.

*First Name and Middle Initial(s)	*Last Name	*Suffix (eg, Jr, III)	Academic Degrees	Institution	Location (city, state/province, country)	Role or Contribution, eg, chair, principal investigator	Group (if more than 1 Group listed in the byline) and/or Subgroup (eg, Steering Committee)
Jan	Vynckier		MD	Onze-Lieve-Vrouw Ziekenhuis	Aalst, Belgium	Principle investigator	
Jelle	Demeestere		MD	KU Leuven, Department of Neurosciences, Experimental Neurology; University Hospitals Leuven, Department of Neurology	Leuven, Belgium	Sub-investigator	
Jens	Offermann		MD	Department of Neurology, Vivantes Hospital Neukölln	Berlin, Germany	Sub-investigator	
Jeyaraj Durai	Pandian		MD DM FRCP	Christian Medical College and Hospital	Ludhiana, Punjab, India	Principle investigator	
João André	Sousa		MD	Department of Neurology, Centro Hospitalar e Universitário de Coimbra,	Coimbra, Portugal	Sub-investigator	
João Pedro	Marto		MD	Department of Neurology, Hospital de Egas Moniz, Centro Hospitalar Lisboa Ocidental	Lisbon, Portugal	Principle investigator	
João	Sargento-Freitas		MD, PhD	Department of Neurology, Centro Hospitalar e Universitário de Coimbra	Coimbra, Portugal	Principle investigator	
Jochen	Vehoff		MD	Department of Neurology, Cantonal Hospital St. Gallen	St. Gallen, Switzerland	Principle investigator	
Johann	Pelz		MD	Stroke Department, University Hospital of North Durham	Durham, United Kingdom	Sub-investigator	
John J.	McCabe		PhD	Stroke Clinical Trials Network Ireland	Dublin, Ireland	Sub-investigator	
Joseph	Harbison		MD	St James's Hospital	Dublin, Ireland	Sub-investigator	
Joshua	Mbroh		MD, MSc	Department of Neurology & Stroke Tübingen University	Tübingen, Germany	Study nurse / trial coordinator	
Judith	Wagner		MD, MA, MHBA	Department of Neurology, Evangel. Krankenhaus Gelsenkirchen, Academic Hospital University Essen-Duisburg<br>Department of Neurology 1, Kepler University Hospital, Johannes Kepler University	Gelsenkirchen, Germany<br>Linz, Austria	Principle investigator	
Julien	Niederhauser		MD	Stroke Unit, Groupement Hospitalier de l'Ouest Lémanique	Nyon, Switzerland	Principle investigator	
Jussi	Sipilä		MD, PhD	Department of Neurology, North Karelia Central Hospital<br>Clinical Neurosciences, University of Turku	Joensuu, Finland<br>Turku, Finland	Principle investigator	
Kanta	Tanaka		PhD	Department of Cerebrovascular Medicine, National Cerebral and Cardiovascular Center	Osaka, Japan	Sub-investigator	
Karthika	Rani		BDS, MPH	Division of Stroke, Department of Neurology, Amrita Institute of Medical Sciences,	Kochi, India	Study nurse / trial coordinator	
Katarina	Klimcikova		MD	Department of Neurology, L. Pasteur University Hospital Kosice	Kosice, Slovakia	Sub-investigator	
Kerry	Smith			Southmead Hospital, North Bristol NHS Trust	Bristol, United Kingdom	Study nurse / trial coordinator	
Klaudia	Soltesova		MD	Department of Neurology, L. Pasteur University Hospital Kosice	Kosice, Slovakia	Sub-investigator	
Kosmas	Macha		MD	Department of Neurology, Universitätsklinikum Erlangen, Friedrich-Alexander University Erlangen-Nürnberg	Erlangen, Germany	Sub-investigator	
Kosuke	Matzusono		MD, PhD	Division of Neurology, Department of Medicine, Jichi Medical University	Tochigi, Japan	Sub-investigator	
Kristina	Szabo		MD	Department of Neurology, Medical Faculty Mannheim, University of Heidelberg	Heidelberg, Germany	Sub-investigator	

*First name, last name, and suffix (if applicable) are required and will appear in PubMed.

*First Name and Middle Initial(s)	*Last Name	*Suffix (eg, Jr, III)	Academic Degrees	Institution	Location (city, state/province, country)	Role or Contribution, eg, chair, principal investigator	Group (if more than 1 Group listed in the byline) and/or Subgroup (eg, Steering Committee)
Laetitia	Yperzeele		MD, PhD	NeuroVascular Center, Stroke Unit Antwerp, Department of Neurology, Antwerp University Hospital, Belgium, Translational Neurosciences, Faculty of Medicine and Health Sciences, University of Antwerp,	Antwerp, Belgium	Sub-investigator	
Lars	Alteheld		MD	Department of Neurology, Oslo University Hospital	Oslo, Norway	Principle investigator	
Lars	Kellert		MD	Department of Neurology, University Hospital, Ludwig Maximilians University of Munich	Munich, Germany	Principle investigator	
Liam	Healy		PhD	Cork University Hospital	Cork, Ireland	Principle investigator	
Liqun	Zhang		MD, PhD	Neurology Department, St George's University Hospital	London, United Kingdom	Principle investigator	
Loraine	Fisch		MD	Stroke Unit, Groupement Hospitalier de l'Ouest Lémanique	Nyon, Switzerland	Sub-investigator	
Luana	Gentile		MD	IRCCS Istituto delle Scienze Neurologiche di Bologna, Department of Neurology and Stroke Centre, Maggiore Hospital	Bologna, Italy	Sub-investigator	
Ludwig	Schelosky		MD	Department of Neurology, Cantonal Hospital Münsterlingen	Münsterlingen, Switzerland	Principle investigator	
Lukas	Kellermair		MD, PhD	Department of Neurology 2, Kepler University Hospital GmbH, Johannes Kepler University Linz	Linz, Austria	Sub-investigator	
Lukuman	Gbadamosh		MBBS, FRCP	Royal United Hospital Bath NHS Foundation Trust	Bath, United Kingdom	Principle investigator	
Lynn	Dixon		RN	South Tees Hospitals NHS Foundation Trust	Middlesbrough, United Kingdom	Study nurse / trial coordinator	
Makoto	Nakajima		MD, PhD	Department of Neurology, Graduate School of Medical Sciences, Kumamoto University	Kumamoto, Japan	Principle investigator	
Manabu	Inoue		MD, PhD	Department of Cerebrovascular Medicine, National Cerebral and Cardiovascular Centre	Osaka, Japan	Sub-investigator	
Manju	Krishnan		MD	Stroke Unit, Morriston Hospital, Swansea Bay University Local Health Board	Swansea, United Kingdom	Principle investigator	
Manuel	Bolognese		MD	Centre of Neurology, Cantonal Hospital of Lucerne	Lucerne, Switzerland	Principle investigator	
Marcel	Arnold		MD	Department of Neurology, University Hospital Bern, University of Bern	Bern, Switzerland	Sub-investigator	
Maria Giulia	Mosconi		MD	Stroke Unit – Internal, Vascular and Emergency Medicine, Santa Maria della Misericordia Hospital University of Perugia,	Perugia, Italy	Sub-investigator	
Marianne	Altmann		MD, PhD	Department of Neurology, Akershus University Hospital<br>Institute of Clinical Medicine, University of Oslo	Lørenskog, Norway<br>Nordbyhagen, Norway	Sub-investigator	
Marie	Lang		MD, PhD	Department of Neurology, Medical University of Vienna	Vienna, Austria	Sub-investigator	
Marios	Psychogios		MD	Department of Neuroradiology, University Hospital Basel and University of Basel	Basel, Switzerland	Associate	
Marjaana	Tiainen		PhD	Department of Neurology, Helsinki University Hospital and University of Helsinki	Helsinki, Finland	Principle investigator	
Mark	Barber		MD	University Hospital Monklands, Airdrie	Lanarkshire, United Kingdom	Principle investigator	
Markus	Arnold		MD	Department of Neurology, University Hospital Zurich	Zurich, Switzerland	Sub-investigator	

Supplemental Online Content: Nonauthor Collaborators
*First name, last name, and suffix (if applicable) are required and will appear in PubMed.

*First Name and Middle Initial(s)	*Last Name	*Suffix (eg, Jr, III)	Academic Degrees	Institution	Location (city, state/province, country)	Role or Contribution, eg, chair, principal investigator	Group (if more than 1 Group listed in the byline) and/or Subgroup (eg, Steering Committee)
Markus	Kneihsl		MD, PhD	Department of Neurology, Medical University of Graz; Department of Radiology, Division of Neuroradiology, Vascular and Interventional Radiology, Medical University of Graz	Graz, Austria	Sub-investigator	
Marta	Magriço		MD	Department of Neurology, Hospital de Egas Moniz, Centro Hospitalar Lisboa Ocidental	Lisbon, Portugal	Sub-investigator	
Martin	Müller		MD, PhD	Centre of Neurology, Cantonal Hospital of Lucerne	Lucerne, Switzerland	Sub-investigator	
Mary	Joan MacLeod		PhD	University of Aberdeen, Division of Applied Medicine	Aberdeen, United Kingdom	Principle investigator	
Matthias	Greulich		MD	Department of Neurology, Cantonal Hospital Winterthur	Winterthur, Switzerland	Principle investigator	
Matthieu Pierre	Rutgers		MD	Neurology Department and Stroke Unit of Europe Hospitals	Brussels, Belgium	Principle investigator	
Maximilian	Schell		MD	Department of Neurology, University Medical Centre Hamburg-Eppendorf	Hamburg, Germany	Sub-investigator	
Melissa	Garcia-Pons		MD	Shaare Zedek Medical Centre, Hebrew University	Jerusalem, Israel	Sub-investigator	
Mette	Pøhner Skahjem			Department of Neurology, Drammen Hospital	Drammen, Norway	Sub-investigator	
Michael	Haley		MB BCh	Weston General Hospital, Weston-Super-Mare	Somerset, United Kingdom	Principle investigator	
Michael	Marnane		MB PhD	Neurology Department, Mater Misericordiae University Hospital	Dublin, Ireland	Sub-investigator	
Milan	Vosko		MD, PhD, FESO	Department of Neurology 2, Kepler University Hospital GmbH, Johannes Kepler University Linz	Linz, Austria	Sub-investigator	
Miroslav	Mako		MD	Department of Neurology, Faculty Hospital Trnava<br>Jessenius Faculty of Medicine, Martin, Comenius University	Trnava, Slovakia<br>Bratislava, Slovakia	Principle investigator	
Naren	Polavarapu		MD	Lalitha Super Specialities Hospital	Guntur, India	Sub-investigator	
Nicolas	Martinez-Majander		MD, PhD	Department of Neurology, Helsinki University Hospital and University of Helsinki	Helsinki, Finland	Sub-investigator	
Nicole	Del Gaudio		MD	Neurology Department and Stroke Unit of Europe Hospitals	Brussels, Belgium	Sub-investigator	
Nicoletta G.	Caracciolo		MD	Department of Human Neurosciences, University La Sapienza	Rome, Italy	Sub-investigator	
Nils	Peters		MD	Stroke Center, Hirslanden Clinic	Zurich, Switzerland	Principle investigator	
Niranjan	Mahajan		MD, DM	NH Institute of Neuroscience	Bangalore, India	Sub-investigator	
Norbert	Silimon		MD	Department of Neurology, University Hospital Bern, University of Bern	Bern, Switzerland	Sub-investigator	
Oezguer A.	Onur		MD	Department of Neurology, Faculty of Medicine and University Hospital Cologne, University of Cologne	Cologne, Germany	Principle investigator	
Ole	Morten Rønning		MD, PhD	Department of Neurology, Akershus University Hospital<br>Institute of Clinical Medicine, University of Oslo	Lørenskog, Norway<br>Nordbyhagen, Norway	Sub-investigator	
Peter	Ringleb		MD	Department of Neurology, Heidelberg University Hospital	Heidelberg, Germany	Sub-investigator	
Peter	Slade		Dr.	Stroke Unit, Morriston Hospital, Swansea Bay University Local Health Board	Swansea, United Kingdom	Sub-investigator	
Peter	Vanacker		MD PhD	Department of Neurology, AZ Groeninge Kortrijk	Kortrijk, Belgium	Principle investigator	
Philippe	Desfontaines		MD	Department of Neurology, Comprehensive Stroke Unit, CHC MontLégia Hospital	Liège, Belgium	Principle investigator	

Supplemental Online Content: Nonauthor Collaborators
*First name, last name, and suffix (if applicable) are required and will appear in PubMed.

*First Name and Middle Initial(s)	*Last Name	*Suffix (eg, Jr, III)	Academic Degrees	Institution	Location (city, state/province, country)	Role or Contribution, eg, chair, principal investigator	Group (if more than 1 Group listed in the byline) and/or Subgroup (eg, Steering Committee)
Priya	Nair		MD, MRCP	Perth Royal Infirmary, NHS Tayside	Perth, United Kingdom	Principle investigator	
Rados	Marian		MD	Department of Neurology, University Hospital Tulln	Tulln an der Donau, Austria	Sub-investigator	
Rajsrinivas	Parthasarathy		MRCP	Artemis Hospital	Gurgaon, Haryana, India	Principle investigator	
Rea	Hidalgo			Glan Clwyd Hospital, Betsi Cadwaladr University Local Health Board	Rhyl, United Kingdom	Study nurse / trial coordinator	
Riona	Mulcahy		MD	University Hospital Waterford	Waterford, Ireland	Principle investigator	
Risa	Kato		MD	Department of Neurology, Kansai Medical University	Hirakata, Japan	Sub-investigator	
Rohit	Bhatia		MD, DM	Department of Neurology, All India Institute of Medical Sciences	New Delhi, India	Sub-investigator	
Ronan	Collins		MD	Tallaght University Hospital	Dublin, Ireland	Sub-investigator	
Ronen R.	Leker		MD, FESO, FAHA	Hadassah-Hebrew University Medical Center	Jerusalem, Israel	Principle investigator	
Roni	Eichel		MD	Shaare Zedek Medical Center, Hebrew University	Jerusalem, Israel	Principle investigator	
Sabine	Fenzl		MD	University Institute of Diagnostic and Interventional Neuroradiology, Inselspital, Bern University Hospital, University of Bern	Bern, Switzerland	Sub-investigator	
Ryota	Tanaka		MD, PhD	Division of Neurology, Department of Medicine, Jichi Medical University	Tochigi, Japan	Sub-investigator	
Samer Al	Hussayni Husseini		MD	South Tees Hospitals NHS Foundation Trust	Middlesborough, United Kingdom	Principle investigator	
Sandra	Clarke		MSc	Department of Neurology, Cantonal Hospital of Aarau	Aarau, Switzerland	Study nurse / trial coordinator	
Sapna Erat	Sreedharan		MD, DM	Sree Chitra Tirunal Institute for Medical Sciences and Technology	Kerala, India	Sub-investigator	
Sarah	Ostanek		RGN	Glan Clwyd Hospital, Betsi Cadwaladr University Local Health Board	Rhyl, United Kingdom	Study nurse / trial coordinator	
Sellimi	Amina		MD	University Hospital Saint-Luc Brussels	Brussels, Belgium	Sub-investigator	
Seraina	Beyeler		PhD	Department of Neurology, University Hospital Bern, University of Bern	Bern, Switzerland	Sponsor - Trial Team	
Sharon	Storton			Acute Stroke Unit, Morriston Hospital, Swansea Bay University Health Board	Swansea, Wales	Study nurse / trial coordinator	
Shigeru	Fujimoto		MD, PhD	Division of Neurology, Department of Medicine, Jichi Medical University,	Tochigi, Japan	Principle investigator	
Silja	Räty		MD, PhD	Department of Neurology, Helsinki University Hospital and University of Helsinki	Helsinki, Finland	Sub-investigator	
Simon	Fandler-Höfler		MD, PhD	Department of Neurology, Medical University of Graz	Graz, Austria	Sub-investigator	
Sofia	Galego		MD	Stroke Unit, Lisbon Central University Hospital	Lisbon, Portugal	Sub-investigator	
Sohei	Yoshimura		MD, PhD	Department of Cerebrovascular Medicine, National Cerebral and Cardiovascular Center	Osaka, Japan	Sub-investigator	
Soichiro	Matsubara		MD, PhD	Graduate School of Medical Sciences, Kumamoto University	Kumamoto, Japan	Sub-investigator	
Stefan	Greisenegger		MD	Department of Neurology, Medical University of Vienna	Vienna, Austria	Sub-investigator	
Stefan	Oberndorfer		MD	Department Neurology, University Clinic St Pölten, Karl Landsteiner private University for Health Sciences	St. Poelten, Austria	Principle investigator	

Supplemental Online Content: Nonauthor Collaborators
*First name, last name, and suffix (if applicable) are required and will appear in PubMed.

*First Name and Middle Initial(s)	*Last Name	*Suffix (eg, Jr, III)	Academic Degrees	Institution	Location (city, state/province, country)	Role or Contribution, eg, chair, principal investigator	Group (if more than 1 Group listed in the byline) and/or Subgroup (eg, Steering Committee)
Sucharita	Ray		MD, DM	Department of Neurology, Postgraduate Institute of Medical Education and Research	Chandigarh, India	Sub-investigator	
Susanne	Renaud		MD, PhD	Division of Neurology, Neuchatel Hospital Network	Neuchatel, Switzerland	Sub-investigator	
Susanne	Riebau		MD	Department of Neurology, Neurovascular Center, University of Luebeck	Luebeck, Germany	Sub-investigator	
Sven	Poli		MD	Department of Neurology & Stroke, Tübingen University	Tübingen, Germany	Principle investigator	
Svetlana	Politz			Department of Neurology, Cantonal Hospital Münsterlingen	Münterlingen, Switzerland	Study nurse / trial coordinator	
Sylvan J.	Albert		MD, MSc	Stroke Unit, Cantonal Hospital Graubünden	Graubünden, Switzerland	Principle investigator	
Takenobu	Kunieda		MD, PhD	Department of Neurology, Kansai Medical University	Hirakata, Japan	Sub-investigator	
Takeo	Sato		MD	Department of Neurology, Jikei University School of Medicine	Tokyo, Japan	Sub-investigator	
Takeshi	Yoshimoto		MD, PhD	Department of Neurology, National Cerebral and Cardiovascular Center	Osaka, Japan	Sub-investigator	
Tal	Anjum		FRCP, MSc, MBBS	Acute Stroke Unit, Morriston Hospital, Swansea Bay University Health Board	Swansea, Wales	Sub-investigator	
Tatjana	Pap		MD	Department of Neurology, Medical Faculty Mannheim, University of Heidelberg	Heidelberg, Germany	Sub-investigator	
Telma	Costa		BSN	Royal United Hospital Bath NHS Foundation Trust	Bath, United Kingdom	Study nurse / trial coordinator	
Teresa	Pinho e Melo		MD	Department of Neurology, Hospital de Santa Maria	Lisbon, Portugal	Principle investigator	
Thomas	Iype		MD, DM	Government Medical College Thiruvananthapuram	Kerala, India	Principle investigator	
Tim	Cassidy		FRCP	St Vincent’s University Hospital	Dublin, Ireland	Principle investigator	
Tim J.	Von Oertzen		MD, FRCP	Department of Neurology , Kepler University Hospital, Johannes Kepler University	Linz, Austria	Sub-investigator	
Timo	Kahles		MD	Department of Neurology, Cantonal Hospital Aarau	Aarau, Switzerland	Sub-investigator	
Toni	Danilo		MD, PhD	Department of Human Neurosciences, University La Sapienza	Rome, Italy	Principle investigator	
Torstein	Spetalen		MD	Department of neurology, Drammen Hospital	Drammen, Norway	Principle investigator	
Turgut	Tatlisumak		MD, PhD	Department of Clinical Neuroscience, Institute of Neuroscience and Physiology, Sahlgrenska Academy at University of Gothenburg	Gothenburg, Sweden	CEC chair	
Veerle	De Herdt		MD, PhD	Department of Neurology, Gent University Hospital	Gent, Belgium	Sub-investigator	
Victoria	Borisova		MD	Department of Neurology, Cantonal Hospital of Aarau	Aarau, Switzerland	Study nurse / trial coordinator	
Vijaya	Pamidimukkala		MD, DM	Lalitha super Specialities Hospital	Guntur, India	Principle investigator	
Vikram	Huded		MD, DM	NH Institute of Neuroscience	Bangalore, India	Principle investigator	
Vipul	Gupta		MD	Artemis Hospital	Gurgaon, Haryana, India	Sub-investigator	
Vishav	Kumar		MSc	Christian Medical College and Hospital	Ludhiana, Punjab, India	Study nurse / trial coordinator	
Vivek	Nambiar		MD, DM	Division of Stroke, Department of Neurology, Amrita Institute of Medical Sciences	Kochi, India	Principle investigator	
Waltraud	Pfeilschifter		MD	Department of Neurology, Goethe-University Hospital Frankfurt	Frankfurt, Germany	Principle investigator	

Supplemental Online Content: Nonauthor Collaborators
*First name, last name, and suffix (if applicable) are required and will appear in PubMed.

*First Name and Middle Initial(s)	*Last Name	*Suffix (eg, Jr, III)	Academic Degrees	Institution	Location (city, state/province, country)	Role or Contribution, eg, chair, principal investigator	Group (if more than 1 Group listed in the byline) and/or Subgroup (eg, Steering Committee)
Wendy	Stoop		MSc	Department of Neurology, Gent University Hospital	Gent, Belgium	Study nurse / trial coordinator	
Yasuyuki	Iguchi		MD, PhD	Department of Neurology, Jikei University School of Medicine	Tokyo, Japan	Principle investigator	
Yngve	Müller Seljeseth		MD	Aalesund Hospital, Helse More og Romsdal Health Trust	Aalesund, Norway	Principle investigator	
Youssif	Abousleiman		MD	Neurology Department, St George's University Hospital	London, UK	Sub-investigator	
Yusuke	Yakushiji		MD, PhD	Department of Neurology, Kansai Medical University	Hirakata, Japan	Principle investigator	
Zoltan	Pencz		MD	Royal Stoke Universtiy Hospital, University Hospitals of North Midlands NHS Trust	Stoke-on-Trent, United Kingdom	Principle investigator	
