# Supplementary material for: Net Benefit of Early Anticoagulation for Stroke With Atrial Fibrillation: Post Hoc Analysis of the ELAN Randomized Clinical Trial
Source: JAMA Netw Open. 2025 Jan 28;8(1):e2456307. doi: 10.1001/jamanetworkopen.2024.56307 (PMC11775740; doi:10.1001/jamanetworkopen.2024.56307)
Supplement: Supplement 4. — Data Sharing Statement [file jamanetwopen-e2456307-s004.pdf]

Data Sharing Statement

Polymeris. Net Benefit of Early Anticoagulation for Stroke With Atrial Fibrillation. *JAMA Netw Open*. Published January 28, 2025. doi:10.1001/jamanetworkopen.2024.56307

Data
Additional Information: Trial Registry: clinicaltrials.gov URL: https://www.clinicaltrials.gov/study/NCT03148457 Registration number: NCT03148457
Data available: Yes
Data types: Deidentified participant data
How to access data: Fully anonymised data may be made available after submitting a research and statistical analysis plan to the corresponding author (urs.fischer@insel.ch), subject to approval by the trial's leadership.
When available: beginning date: 01-01-2026, end date: 01-01-2027

Supporting Documents
Document types: None

Additional Information
Who can access the data: researchers whose proposed use of the data has been approved
Types of analyses: Types of analyses: For a research project
Mechanisms of data availability: after approval of a proposal by the steering board and with a signed data access agreement
Any additional restrictions: Final decision to share data lies with the steering board of the trial
